# Impaired GAPDH-induced mitophagy contributes to the pathology of Huntington’s disease

**DOI:** 10.15252/emmm.201505256

**Published:** 2015-08-12

**Authors:** Sunhee Hwang, Marie-Hélène Disatnik, Daria Mochly-Rosen

**Affiliations:** Department of Chemical and Systems Biology, Stanford University School of MedicineStanford, CA, USA

**Keywords:** glyceraldehyde-3-phosphate dehydrogenase, Huntington’s disease, mitochondria, mitophagy, polyglutamine repeats

## Abstract

Mitochondrial dysfunction is implicated in multiple neurodegenerative diseases. In order to maintain a healthy population of functional mitochondria in cells, defective mitochondria must be properly eliminated by lysosomal machinery in a process referred to as mitophagy. Here, we uncover a new molecular mechanism underlying mitophagy driven by glyceraldehyde-3-phosphate dehydrogenase (GAPDH) under the pathological condition of Huntington’s disease (HD) caused by expansion of polyglutamine repeats. Expression of expanded polyglutamine tracts catalytically inactivates GAPDH (iGAPDH), which triggers its selective association with damaged mitochondria in several cell culture models of HD. Through this mechanism, iGAPDH serves as a signaling molecule to induce direct engulfment of damaged mitochondria into lysosomes (micro-mitophagy). However, abnormal interaction of mitochondrial GAPDH with long polyglutamine tracts stalled GAPDH-mediated mitophagy, leading to accumulation of damaged mitochondria, and increased cell death. We further demonstrated that overexpression of inactive GAPDH rescues this blunted process and enhances mitochondrial function and cell survival, indicating a role for GAPDH-driven mitophagy in the pathology of HD.

## Introduction

Huntington’s disease (HD), affecting ∼5–10 per 100,000 people in the Western world (Ross & Tabrizi, 2011; Mochly-Rosen *et al*, 2014), is a progressive neurodegenerative disease caused by a genetic mutation in the huntingtin gene, leading to the production of an expanded polyglutamine stretch in the encoded protein. Mutant huntingtin protein likely affects a wide range of cellular pathways and functions by abnormally interacting with a variety of proteins and intracellular organelles, thus contributing to the pathology underlying HD (Michalik & Van Broeckhoven, 2003; Li & Li, 2004). Affected HD patients usually carry more than 35 repeats in their mutant huntingtin as compared to 16–20 repeats in the normal population (Labbadia & Morimoto, 2013). HD patients develop various physical and psychiatric disturbances over 10–20 years after disease onset and die due to complications from the disease (Zuccato *et al*, 2010). Although the mutation in the huntingtin gene was identified more than 20 years ago (HD Collaborative Research Group, 1993), only symptomatic management (Novak & Tabrizi, 2010; Ross & Tabrizi, 2011) is currently available to treat the disease. Thus, it is important to identify the molecular mechanisms leading to the pathology associated with mutant huntingtin in HD and develop therapeutics targeting them.

Mitochondrial dysfunction has been implicated in the pathology of HD because neurons, with high-energy demands, are dependent on mitochondria as a source of energy production to maintain their functions (Costa & Scorrano, 2012). Mitochondria actively move from the neuronal soma to the axon and dendrites and constantly undergo repeated cycles of fusion and fission in a process regulated by the dynamin family of GTPases (guanosine triphosphatases) (Chan, 2012). However, mitochondrial fusion and fission, collectively referred to as mitochondrial dynamics, are significantly perturbed and imbalanced in HD (Shirendeb *et al*, 2011; Song *et al*, 2011), leading to accumulation of fragmented, damaged mitochondria and increased levels of oxidative stress in cells. We recently found that inhibition of excessive mitochondrial fission (but not physiological fission) by a selective inhibitor of the mitochondrial fission protein, dynamin-related protein 1 (Drp1), prevents mutant huntingtin-induced pathology *in vitro* and *in vivo* (Guo *et al*, 2013), suggesting that mitochondrial dynamics is a potential therapeutic target for HD.

The cycle of fusion and fission is further closely coordinated with mitochondrial autophagy or mitophagy, a process of selective elimination of damaged mitochondria by autophagic machinery. Through mitophagy, damaged mitochondria are sequestered into double membrane-bound autophagosomes and subsequently catabolized via lysosomal degradation (Youle & van der Bliek, 2012; Kornfeld *et al*, 2015). To maintain metabolic homeostasis and viability of cells, mitochondria can also be degraded non-selectively, together with other cytosolic contents, when cells are deprived of nutrients (Youle & Narendra, 2011). A number of molecular pathways regulating mitophagy in different types of cells from various organisms have been reported, including autophagy-related 32 (Atg32) in yeast, NIP1-like protein X (NIX) in red blood cells, and phosphatase and tension homolog-induced putative kinase 1 (PINK1) and Parkin in many mammalian cells (Schweers *et al*, 2007; Okamoto *et al*, 2009; Matsuda *et al*, 2010). In HD and in other neurodegenerative diseases, abnormalities at different phases of mitophagy (Martinez-Vicente *et al*, 2010; Wong & Holzbaur, 2014) have been proposed to contribute to disease progression, albeit through unknown mechanisms.

In this study, we investigated the molecular mechanism by which removal of damaged mitochondria is impaired in HD. Our group has previously identified a new form of micro-mitophagy in which glyceraldehyde-3-phosphate dehydrogenase (GAPDH) associates with damaged mitochondria under oxidative stress induced by ischemia–reperfusion injury in hearts and promotes direct uptake of damaged mitochondria into the lysosomal structure that is composed of hybrid organelles of lysosomes and late endosomes. This micro-mitophagy occurs independently of the catalytic/metabolic activity of GAPDH (Yogalingam *et al*, 2013). Here, we sought to explore whether and how this GAPDH-driven mitophagy is regulated in HD. To date, many proteins interacting with and/or affected by mutant huntingtin have been reported (Li & Li, 2004), and notably, GAPDH has been identified as one such protein (Burke *et al*, 1996; Wu *et al*, 2007). However, except for a reduced glycolytic activity of GAPDH in fibroblast cells derived from HD patients (Mazzola & Sirover, 2001), other biochemical and physiological consequences of the association of mutant huntingtin and GAPDH have not been examined. We report that expression of mutant huntingtin with expanded polyglutamine repeats negatively regulates GAPDH-driven mitophagy, thus contributing to HD-associated pathology.

## Results

### GAPDH selectively associates with mitochondria in cells expressing expanded polyglutamine repeats

Exon 1 of the huntingtin gene followed by long polyglutamine repeats has been extensively used to study HD, because this fragment is sufficient to form intracellular aggregates, cellular toxicity, and cell death (Paulson *et al*, 1997; Cooper *et al*, 1998; Igarashi *et al*, 1998; Wyttenbach *et al*, 2001; Mochly-Rosen *et al*, 2014). To study the biology of Huntington’s disease in cell culture, we used three different models that express the exon 1 of huntingtin. The first model used was a PC12 cell line (derived from rat pheochromocytoma) that expresses an inducible EGFP-tagged exon 1 of huntingtin with 23 or 74 polyglutamine repeats (Q23 or Q74) (Wyttenbach *et al*, 2001). The expression level of Q23 or Q74 was significantly increased after induction by doxycycline and remained constant from days 2 to 6 (Appendix Fig S1A, left). We observed a higher level of polyglutamine expression in cells with Q74 relative to cells with Q23, which may be due to the resistance of expanded polyglutamine repeats to degradation (Ferrigno & Silver, 2000; Sun *et al*, 2002). Furthermore, more polyglutamine aggregates in cells expressing Q74 were observed by EGFP fluorescence (Appendix Fig S1A, right). We also used a striatal cell line from HD transgenic knock-in mice, expressing a full length of huntingtin containing short (Q7) or long (Q111) polyglutamine repeats. As a third model, we used HD patient-derived fibroblasts (Trettel *et al*, 2000; Wexler *et al*, 2004) and compared them to human fibroblasts derived from healthy controls.

**Figure 1:**
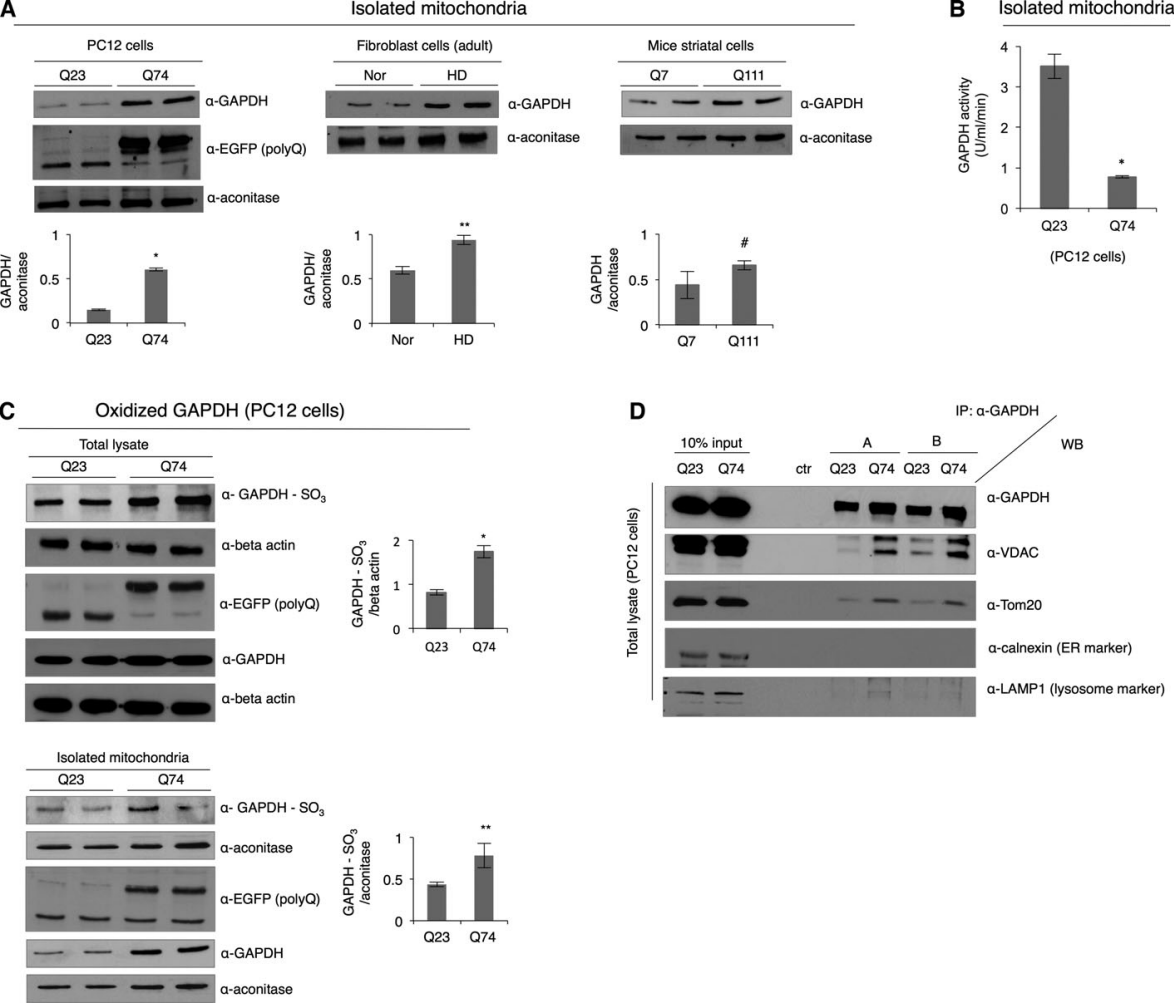
GAPDH selectively associates with mitochondria in cells expressing expanded polyglutamine repeats Representative Western blots showing GAPDH association with mitochondria-enriched fractions in different HD models. The levels of GAPDH were normalized to mitochondrial matrix protein, aconitase. *n *=* *3. *P =* *0.0005; **P =* *0.019; ^#^*P *=* *0.042.
Measurement of GAPDH activity in the mitochondria-enriched fraction isolated from PC12 cells. *n *=* *3. *P =* *0.0005.
Western blots showing oxidized GAPDH in the total lysates of PC12 cells and isolated mitochondria. The levels of oxidized GAPDH were normalized to beta-actin (total lysate) and aconitase (mitochondrial fraction), respectively. *n *=* *2. *P =* *0.023; **P =* *0.039.
Western blot showing co-immunoprecipitation of GAPDH from the total lysates of PC12 cells in RIPA buffer without detergent (Triton X-100). Immunoprecipitated samples were immunoblotted with antibodies against mitochondrial (VDAC and Tom20), endoplasmic reticulum (calnexin), and lysosomal markers (LAMP1). ctr: control. *n *=* *2. Data information: The data are presented as mean ± SEM. Statistical significance was assessed by Student’s *t*-test. Source data are available online for this figure.

Mitophagy is regulated by a number of proteins in response to stress and/or various external insults such as nutrient starvation and oxidative stress (Youle & Narendra, 2011; Kubli & Gustafsson, 2012). Here, we determined whether the novel form of micro-mitophagy, whereby catalytically inactive GAPDH mediates direct uptake of damaged mitochondria into the lysosomal vacuoles (Yogalingam *et al*, 2013), is impaired in HD. The first marker for this form of mitophagy (Yogalingam *et al*, 2013), GAPDH association with the mitochondria, was consistently observed; there was a greater than two-fold increased level of GAPDH in mitochondria-enriched fractions from all the HD models with expanded polyglutamine repeats that we examined, including isolated mitochondria from the brain extracts of the R6/2 HD transgenic mouse model (Fig 1A and Appendix Fig S1B). Furthermore, we noticed that despite the increased amount of GAPDH at the mitochondria from cells with expanded polyglutamine repeats, the catalytic activity of mitochondria-associated GAPDH was drastically lower than in cells expressing short polyglutamine repeats (Fig 1B). We next determined whether the low catalytic activity in cells containing expanded polyglutamine repeats reflects oxidation of catalytically important cysteine (Cys) residues in the active site of GAPDH (Hwang *et al*, 2009; Peralta *et al*, 2015). Using a site-specific anti-GAPDH antibody (α-GAPDH-SO_3_) that detects oxidation of Cys152 in the active site of GAPDH, we found that the level of GAPDH oxidation in the total lysate of PC12 cells with Q74 was almost twice higher than in the lysate with Q23 (Fig 1C, top). Mitochondrial GAPDH in cells expressing Q74 also exhibited a higher level of oxidation as well (Fig 1C, bottom).

We further set out to determine whether GAPDH selectively recognizes and associates with mitochondria in cells expressing expanded polyglutamine repeats. Immunoprecipitation of GAPDH from the PC12 cell extract in the absence of detergent (Triton X-100) showed that in cells expressing Q74, GAPDH co-immunoprecipitated with the mitochondrial marker proteins, VDAC (voltage-dependent anion channel) and Tom20, but not with marker proteins of other organelles such as endoplasmic reticulum (Fig 1D). Lysosomal marker (LAMP1, lysosomal-associated membrane protein 1) was also present in the immunoprecipitates from the extract with Q74, which will be discussed later. Therefore, cellular stress induced by the presence of expanded polyglutamine repeats seems to increase association of GAPDH with mitochondria.

### Expanded polyglutamine repeats cause mitochondrial dysfunction

**Figure 2:**
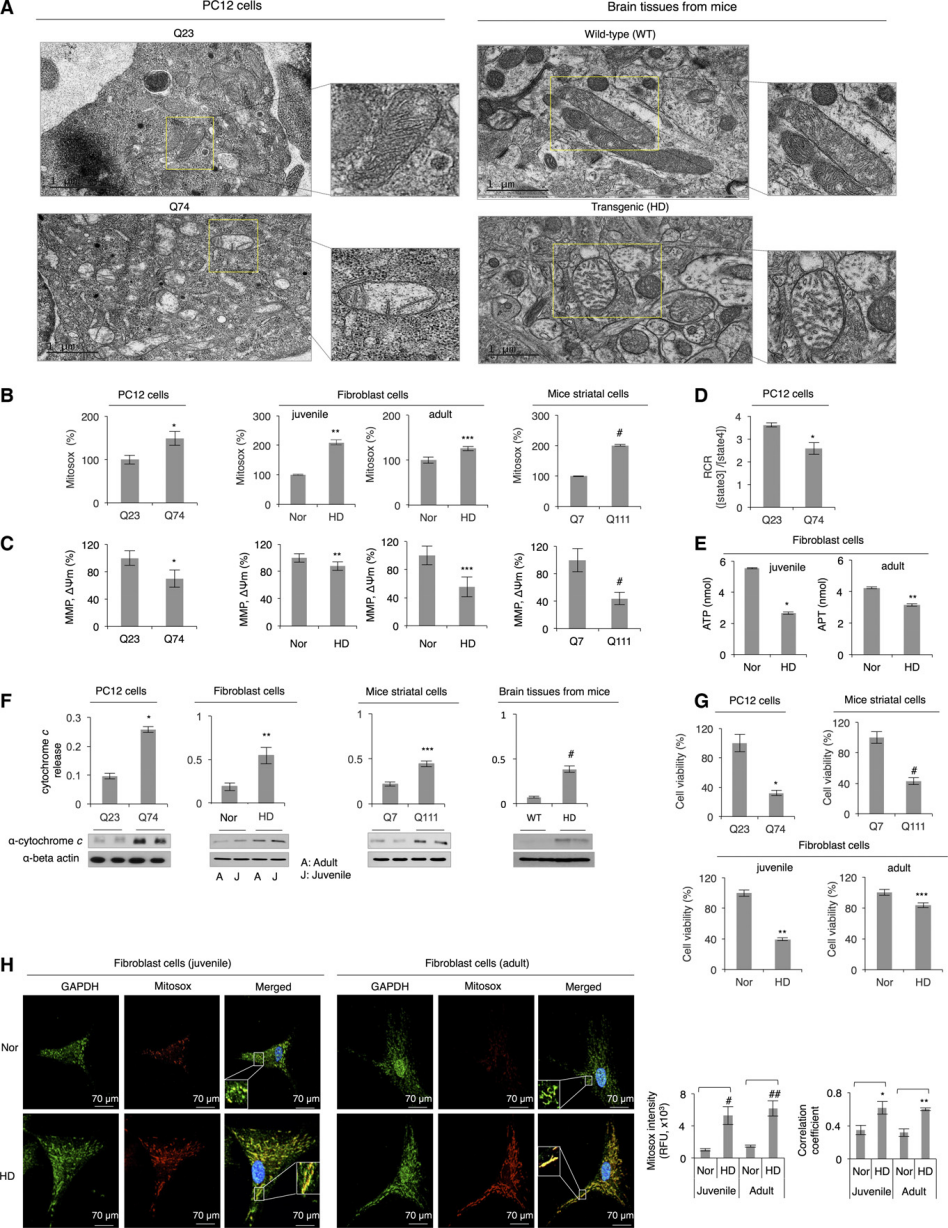
Expanded polyglutamine repeats cause mitochondrial dysfunction Representative electron micrographs showing mitochondrial morphology in PC12 cells expressing Q23 (top panel, left) and Q74 (bottom panel, left) and in brain tissues from wild-type (WT) (top panel, right) and HD transgenic mice (bottom panel, right). An indicated portion of each image (in yellow) is expanded to illustrate the mitochondrial morphology. Scale bars: 1 μm.
Measurement of mitochondrial ROS production (using Mitosox) in different HD models. Results are presented as percent of control [Q23, normal fibroblast (Nor), Q7]. *n *=* *3. *P =* *0.001; **P =* *0.0001; ***P =* *0.0001; ^#^*P *=* *0.0045.
Mitochondrial membrane potential measured by using JC-1 dye in models of HD and control cells (MMP, ΔΨm). *n *=* *3. *P =* *0.008; **P =* *0.044; ***P =* *0.025; ^#^*P *=* *0.005.
Mitochondrial respiratory function in PC12 cells, represented by respiratory control ratio (RCR; [state3]/[state4]). *n *=* *2. *P =* *0.028.
ATP levels in HD patient-derived fibroblast cells. *n *=* *2. *P =* *0.003; **P =* *0.004.
Western blots showing cytochrome *c* release from mitochondria. *n *=* *2. *P =* *0.003; **P =* *0.003; ***P =* *0.01; ^#^*P *=* *0.008.
Cell viability determined by colorimetric assays utilizing WST8 conversion to WST8 formazan in different HD models. *n *=* *3. *P, **P, ***P, ^#^*P *<* *0.005.
Representative immunofluorescence of normal and HD patient-derived fibroblasts stained for GAPDH and mitochondrial ROS with Mitosox. Three independent images per condition were analyzed to calculate Mitosox intensity and the Pearson’s correlation coefficient for quantification of colocalization between GAPDH and mitochondrial ROS. ^#^*P *=* *0.03; ^##^*P *=* *0.02; *P =* *0.006; **P =* *0.005. Images were acquired at 63× magnification, and brightness and contrast of images were adjusted by 50%. Data information: The data are presented as mean ± SEM. Statistical significance was assessed by Student’s *t*-test. Source data are available online for this figure.

To examine whether GAPDH-bound mitochondria in cells expressing expanded polyglutamine repeats are damaged, we measured several key aspects of mitochondrial function. First, we characterized mitochondrial morphology. PC12 cells expressing short polyglutamine repeats (Q23) contained long and tubular-shaped mitochondria with dense cristae, whereas cells expressing expanded polyglutamine repeats (Q74) contained small and swollen mitochondria with distorted cristae (Fig 2A and Appendix Fig S2A). To quantify the change in mitochondrial morphology, mitochondria were traced from TEM micrographs, and their morphology was analyzed as previously described (Picard *et al*, 2013). Specifically, the form factor reflects a degree of mitochondrial branching; roundness is a sphericity index; Feret’s diameter is a measure of the longest distance (μm) between any two points within a mitochondrion; and aspect ratio represents a ratio between the major and minor axes of a mitochondrion, reflecting mitochondrial elongation (Picard *et al*, 2013). Based on this analysis (Appendix Fig S2A), mitochondria in cells with Q74 appeared smaller, shorter, and more round shaped, relative to cells expressing Q23. Smaller and more fragmented mitochondria with reduced mitochondrial interconnectivity were also observed in HD patient-derived fibroblasts as compared to normal fibroblasts (Appendix Fig S2B). We next examined mitochondrial function and found that expression of expanded polyglutamine repeats in PC12 cells, HD patient-derived fibroblasts, and mice striatal neurons all showed a significantly increased ROS production (Fig 2B), as measured by Mitosox, a mitochondrial superoxide indicator. This is consistent with the previous studies (Wyttenbach *et al*, 2002; Hands *et al*, 2011) that protein aggregation of polyglutamine repeats causes free radical production *in vitro* and in cultured cells. Furthermore, mitochondria in cells with expanded polyglutamine repeats were depolarized as indicated by a decrease in mitochondrial membrane potential measured by JC-1 dye, (MMP, ΔΨm: Fig 2C), and mitochondrial respiratory function (Fig 2D) and ATP generation (Fig 2E) were impaired as well. We also observed a significantly increased cytochrome *c* release from mitochondria to the cytosol (a marker of cell apoptosis) in cells with expanded polyglutamine repeats and in brain extracts of HD transgenic mouse model (R6/2) as compared to the level in the respective controls (Fig 2F), together with a considerably reduced cell viability measured by the colorimetric assay (Fig 2G). All these data indicate that mitochondria in cells expressing expanded polyglutamine repeats are most likely damaged and dysfunctional and GAPDH selectively recognizes and associates with these mitochondria. In support of this, GAPDH highly localized with mitochondria-generated ROS in HD patient-derived fibroblast cells that were stained for Mitosox and GAPDH, but not in fibroblasts of healthy controls (Fig 2H).

### Expanded polyglutamine repeats affect elimination of damaged mitochondria by mitophagy

As previously reported, mitochondrial GAPDH induces direct engulfment of damaged mitochondrial into lysosomes for degradation in cardiac myocytes (Yogalingam *et al*, 2013). To determine how GAPDH-mediated mitophagy is regulated in HD and the mechanism by which expanded polyglutamine repeats affect mitochondrial elimination, we first used an *in vitro* system in which intracellular organelles (particularly lysosomes and mitochondria) are isolated using a density gradient. Eight fractions were collected from the top (1) to the bottom (8) of the gradient and analyzed by Western blot for the presence of lysosomes and mitochondria. To characterize this system, we first utilized mouse embryonic fibroblasts (MEFs) that lack Atg5, a key gene involved in autophagosome formation, and are therefore defective in autophagy (Mizushima *et al*, 2001). Atg5 wild-type (WT) and knockout (KO) MEFs were subjected to serum starvation for 24 h, which triggers autophagy (Li *et al*, 2013). Under this condition, LC3II activation was observed in the Atg5 WT MEFs, but not in the Atg5 KO MEFs (Appendix Fig S3A). In addition, VDAC levels in the Atg5 KO MEFs rather increased after this stress, providing further evidence for a defective autophagy process (Appendix Fig S3A). Previous reports indicated that under stressed conditions, dysfunctional mitochondria accumulate in cells lacking Atg5 gene (Tal *et al*, 2009; Chang *et al*, 2010). Thus, to further demonstrate a process of mitochondrial elimination in those cells, lysosomes and mitochondria isolated from the Atg5 WT or KO MEFs were fractionated by a density gradient. Under normal condition, lysosomes accumulated in the upper layers (mostly in fractions 1–4, Appendix Fig S3B) and mitochondria in the bottom layers (mostly in fractions 4–8, Appendix Fig S3B), as indicated by the presence of lysosomal marker protein, LAMP1, and mitochondrial marker protein, VDAC, respectively. When cells were serum-starved, mitochondria accumulated in the (upper) lysosomal fractions only in the Atg5 KO MEFs and not in the WT MEFs (Appendix Fig S3B), suggesting that when damaged mitochondria are not properly eliminated, they associate with the lysosomal fractions.

**Figure 3:**
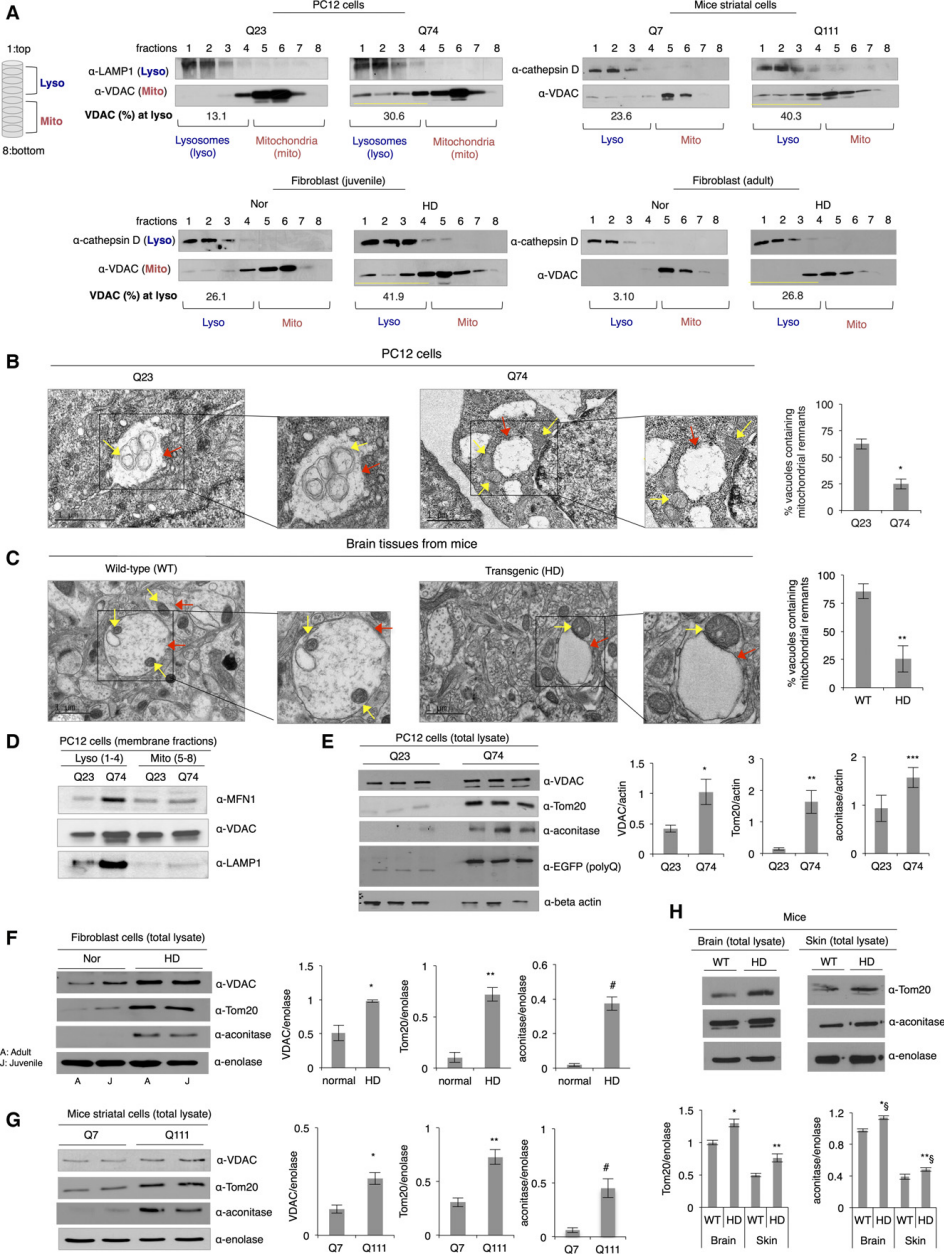
Expanded polyglutamine repeats affect elimination of damaged mitochondria by mitophagy A Representative Western blots showing separation of lysosome- and mitochondria-enriched fractions by a density gradient. Total extracts from different HD models were fractionated by the density gradient. Eight fractions were collected from top (#1) to the bottom (#8). The amount of mitochondria in the lysosomal fractions was presented as a percentage of VDAC present in the entire gradient. *n *=* *3.
B, C Representative electron micrographs of PC12 cells expressing Q23 or Q74 and brain tissues from wild-type (WT) and HD transgenic R6/2 mice showing mitochondrial (as indicated by yellow arrows) association with lysosomal vacuoles (shown by red arrows). The vacuoles containing mitochondrial remnants are presented as a percentage of the total vacuoles counted. Analysis (of six independent images) was carried out by an observer blinded to the experimental conditions. *P <* *0.023; **P <* *0.035. Scale bar: 1 μm.
D Western blot showing association of mitochondrial membrane with lysosomal membrane. The presence of membranes of each organelle was assessed using anti-MFN1, VDAC (as mitochondria markers), and LAMP1 (as a lysosomal marker). *n *=* *3.
E Representative Western blot showing mitochondrial contents (VDAC, Tom20, and aconitase) in the total lysates of PC12 cells with Q23 or Q74. Mitochondrial protein levels were normalized to beta-actin. *n *=* *3. *P =* *0.024; **P =* *0.0008; ***P =* *0.03.
F Representative Western blot showing mitochondrial contents (VDAC, Tom20, and aconitase) in the total lysates of normal fibroblast cells and HD patient-derived fibroblasts. Mitochondrial protein levels were normalized to the cytosolic protein, enolase. *n *=* *3. *P =* *0.008; **P =* *0.002; ^#^*P *=* *0.0008.
G Representative Western blot showing mitochondrial contents (VDAC, Tom20, and aconitase) in the total lysates of mice striatal cells with Q7 and Q111. *n *=* *3. *P =* *0.0008; **P =* *0.0007; ^#^*P *=* *0.001.
H Western blot showing mitochondrial contents (Tom20 and aconitase) in the total lysates of brain tissues from wild-type (WT) and HD transgenic mice. *n *=* *2. *P =* *0.02; **P =* *0.03 *^§^*P *=* *0.01; **^§^*P *<* *0.04. Data information: The data are presented as mean ± SEM. Statistical significance was assessed by Student’s *t*-test. Source data are available online for this figure.

Similarly, lysosome- and mitochondria-enriched fractions from control cells (represented by expression of Q23 in PC12 cells, Q7 in mice striatal neuronal cells, and normal human fibroblasts) were well separated using this density gradient. The mitochondria were segregated to the bottom layers of the gradients (fraction 4(5)–8), as indicated by the presence of VDAC, whereas lysosome-enriched fractions were found mainly at the upper layers of the gradients (fraction 1–4), as shown by the presence of either LAMP1 or cathepsin D (Fig 3A). This suggests that mitochondria undergo basal mitophagy (no accumulation of mitochondria in lysosomal fractions) or alternatively both organelles lie apart from each other. However, in cells containing expanded polyglutamine repeats, similar to the autophagy-defective Atg5 KO MEFs (Appendix Fig S3B), mitochondrial marker was found also in the lysosomal fractions (Fig 3A), suggesting that lighter (empty, damaged, and fragmented) mitochondria accumulated and co-fractionated with upper lysosome-enriched fractions. Consistently, TEM studies support our interpretation of the gradient experiment. Our group previously identified single membrane-bound vacuolated structures that are of a late endosomal and lysosomal origin, participating in elimination of damaged mitochondria, which we referred to as lysosomal-like (LL) structures (Yogalingam *et al*, 2013). We found that damaged mitochondria are directly internalized for degradation under oxidative stress (Yogalingam *et al*, 2013). In addition to observation of double membrane-bound autophagosomes for a process of macro-autophagy (Appendix Fig S3C), we observed single membrane-bound vacuoles, varied in size, in various HD models. In PC12 cells expressing Q23, mitochondria (yellow arrows) were found in the lysosomal vacuoles (red arrows) (Fig 3B), whereas damaged mitochondria were accumulated around the lysosomal vacuoles rather than inside in cells expressing Q74 (Fig 3B). The vacuolar structures in the Q74-containing cells appeared almost empty, and the percent of vacuoles containing mitochondria was almost 2.5 times lower than that in cells with Q23 (Fig 3B). Similarly, in brain tissues from wild-type mice, we observed single membrane-bound vacuoles that contained mitochondria, whereas in samples from HD transgenic mice, we noticed more mitochondria associated with/around or bound to empty vacuoles (Fig 3C). The percent of vacuoles containing mitochondrial remnants in the brain sections of the HD transgenic mice was almost three times lower relative to the wild-type mice (Fig 3C). TEM analysis of isolated mitochondria from PC12 cells with Q23 also showed the same phenotype of mitochondria and mitochondrial remnants in vacuoles, whereas the Q74-containing PC12 cells exhibited mitochondrial accumulation around the vacuoles (Appendix Fig S3D). Supporting these observations, mitochondrial membranes co-fractionated with lysosomal membranes containing a late endosomal marker (Rab9) to a greater extent in samples from cells containing Q74 relative to Q23, as indicated by the presence of outer mitochondrial membrane proteins, mitofusin 1 (MFN1) and VDAC, in the lysosome-enriched membrane fractions (Fig 3D and Appendix Fig S3E). Also as mentioned earlier (Fig 1D), in the absence of detergent (Triton X-100), GAPDH associated with mitochondria only in cells expressing Q74, indicating the selective association of GAPDH with mitochondria in samples where they were shown to be damaged. Additionally, we noted that LAMP1 (lysosomal marker) was also associated with the damaged mitochondria, supporting the association of mitochondria around lysosomes when Q74 was expressed (Fig 1D). Interestingly, the expression levels of LAMP1 and Rab9 were higher in cells with Q74 (Fig 3D and Appendix Fig S3E). In accordance with this, there were three times more single membrane-bound vacuoles formed when Q74 was expressed in PC12 cells, as well as increased levels of TFEB (transcription factor EB), a marker for lysosomal biogenesis, in PC12 cells with Q74 (Appendix Fig S3F). The increase in TFEB may reflect an increase in lysosomal biogenesis in order to meet the cellular demand for degradation capacity under stressed conditions. However, polyglutamine repeats are known to interfere with a TFEB function to impair autophagy, as evidenced in recent study (Cortes *et al*, 2014).

We also observed that the presence of expanded polyglutamine repeats markedly increased the amount of mitochondrial proteins, Tom20, VDAC, and aconitase, in the total extracts from different HD models, as shown in Figure E–H. Noticeably, mitochondrial biogenesis significantly decreased, as examined by the transcription level of a representative mitochondrial DNA encoding the gene, mitochondrial NADH dehydrogenase 2 (a subunit of mitochondrial complex I), in cells with Q74 as compared with control cells expressing Q23 (Appendix Fig S3G). This result supports a previous report showing reduced mitochondrial biogenesis in neurons from patients with moderate-to-severe grade HD (Kim *et al*, 2010; Reddy & Shirendeb, 2012). Taken together, this supports that increased mitochondrial mass was mainly caused by the accumulation of damaged mitochondria in cells. Further, it was neither because of increased cell number nor mitochondrial biogenesis.

However, these data contradict the expectation that association of GAPDH with mitochondria would induce elimination of damaged mitochondria; the increased accumulation of mitochondrial proteins may rather reflect a blunted process of elimination of damaged mitochondria. Thus, GAPDH-bound damaged mitochondria are likely accumulated around the lysosomal structures and/or internalized into them without proper degradation because of impaired lysosomal function in cells by expanded polyglutamine repeats as mentioned above (Appendix Fig S3H).

### Expanded polyglutamine repeats selectively associate with mitochondrial GAPDH, inhibiting GAPDH-induced mitophagy

**Figure 4:**
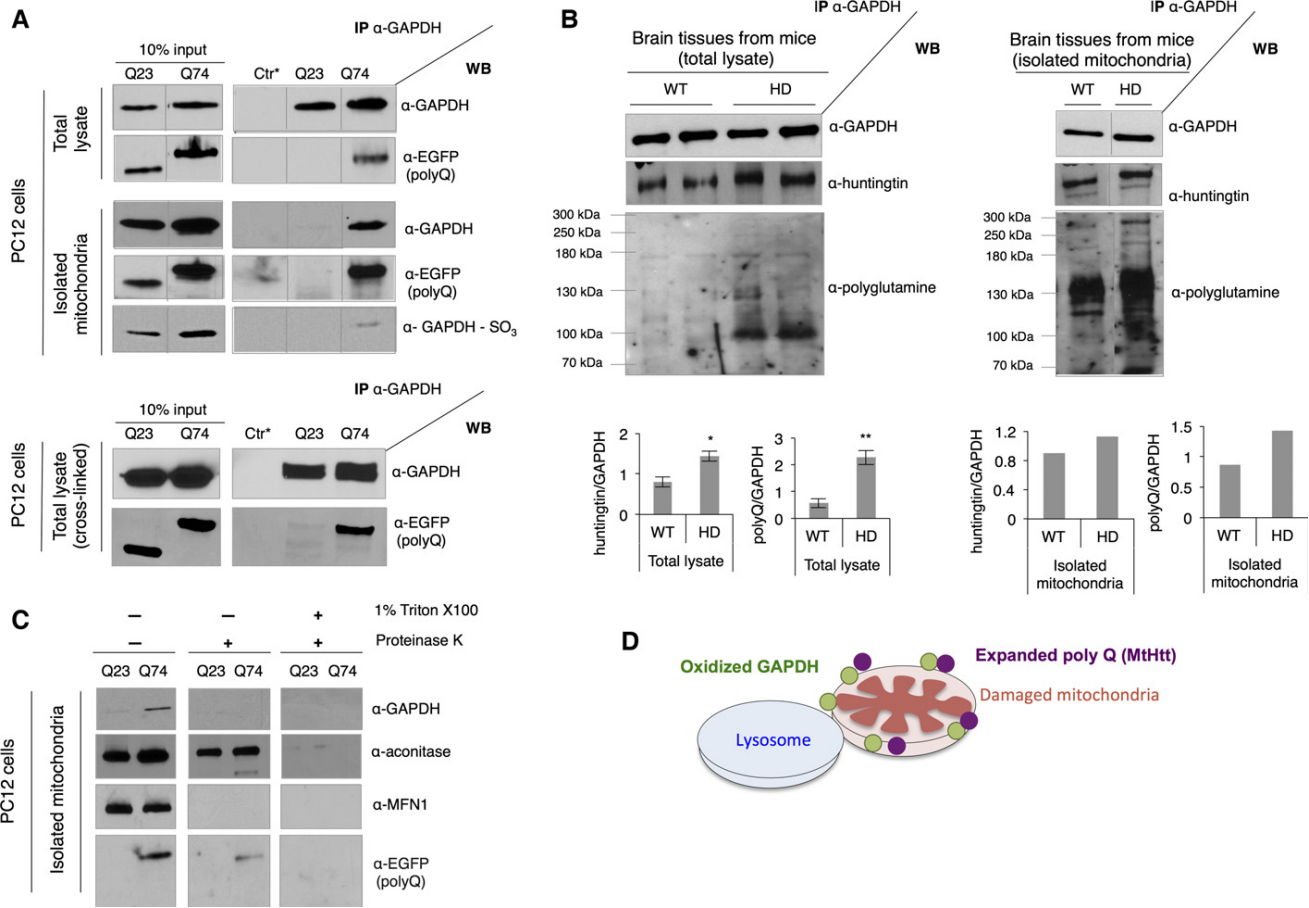
Expanded polyglutamine repeats selectively associate with mitochondrial GAPDH, inhibiting GAPDH-induced mitophagy Western blots showing co-immunoprecipitation of GAPDH from the total lysates and isolated mitochondria of PC12 cells with anti-GAPDH antibody. Anti-EGFP antibody detects the presence of polyglutamine repeats. Bottom panel shows co-immunoprecipitation of GAPDH in the total lysate of PC12 cells treated with formaldehyde to cross-link proteins. Ctr* = control. *n *=* *3.
Western blots showing co-immunoprecipitation of GAPDH from the total lysate (left) and isolated mitochondria (right) of brain tissues of wild-type (WT) and transgenic R6/2 (HD) mice with anti-GAPDH antibody. The presence of huntingtin was examined with anti-huntingtin antibody and anti-polyglutamine antibody, respectively. Co-immunoprecipitated huntingtin or polyglutamine (polyQ) was normalized to GAPDH. Left: *n *=* *2. *P =* *0.03; **P =* *0.02. Right: One representative experiment from two repeats.
Sub-mitochondrial localization of GAPDH and expanded polyglutamine repeats. Isolated mitochondria were left untreated or subjected to the proteinase K treatment in the presence or absence of detergent (Triton X-100). *n *=* *3.
Illustration describing association of oxidized GAPDH (represented by green circles) and expanded polyglutamine repeats (in mutant huntingtin protein (MtHtt) represented by purple circles) at the outer membrane of damaged mitochondria. Data information: The data are presented as mean ± SEM. Statistical significance was assessed by Student’s *t*-test. Source data are available online for this figure.

Because oxidized, inactive GAPDH associated with damaged mitochondria (Fig 1A), yet the amount of mitochondrial proteins in cells expressing expanded polyglutamine repeats was higher than in cells expressing short polyglutamine repeats (Fig 3E–H), we next determined whether GAPDH-driven mitophagy is impaired by presence of expanded polyglutamine repeats. A co-immunoprecipitation experiment demonstrated the association of GAPDH with polyglutamine repeats (represented by the presence of EGFP) in both total lysates and mitochondria-enriched fractions; only Q74 exhibited association with GAPDH (Fig 4A). In particular, Q74-associated mitochondrial GAPDH was shown to be oxidized, indicating that such GAPDH is an inactivated form (Fig 4A). Furthermore, Q74 and GAPDH directly associated with each other, as evidenced in co-immunoprecipitation of GAPDH from the PC12 cells treated with 1% formaldehyde to cross-link the proteins (Fig 4A). Along with this, higher amounts of mutant huntingtin protein from the total extract and isolated mitochondria from brain tissues of HD transgenic mice co-immunoprecipitated with GAPDH relative to the samples from wild-type mice (Fig 4B).

Sub-mitochondrial localization of GAPDH and expanded polyglutamine repeats was further examined using proteinase K, which in the absence of detergent, can only digest exposed proteins, localized on the outer mitochondrial membrane. As expected, proteinase K digested only MFN1, an outer mitochondrial membrane protein, and not mitochondrial matrix proteins (represented by aconitase) when incubated with mitochondria isolated from PC12 cells expressing Q23 or Q74, in the absence of detergent. Similarly, both GAPDH and expanded polyglutamine repeats were degraded in the absence of detergent (Fig 4C), indicating that they are mainly co-localized to the outer mitochondrial membrane. Some of expanded polyglutamine repeats may be inside the mitochondria (some Q74 remained intact after the proteinase K treatment). The treatment with Triton X-100 served as a control to show the sensitivity of the protein degradation to proteinase K. Taken together, we confirmed that expanded polyglutamine repeats bound to mitochondrial GAPDH at the outer mitochondrial membrane, potentially disrupting GAPDH-driven trafficking of damaged mitochondria to the lysosomal system (Fig 4D).

### Overexpression of inactive GAPDH rescues polyglutamine-induced blunted mitophagy and recovers mitochondrial function

**Figure 5:**
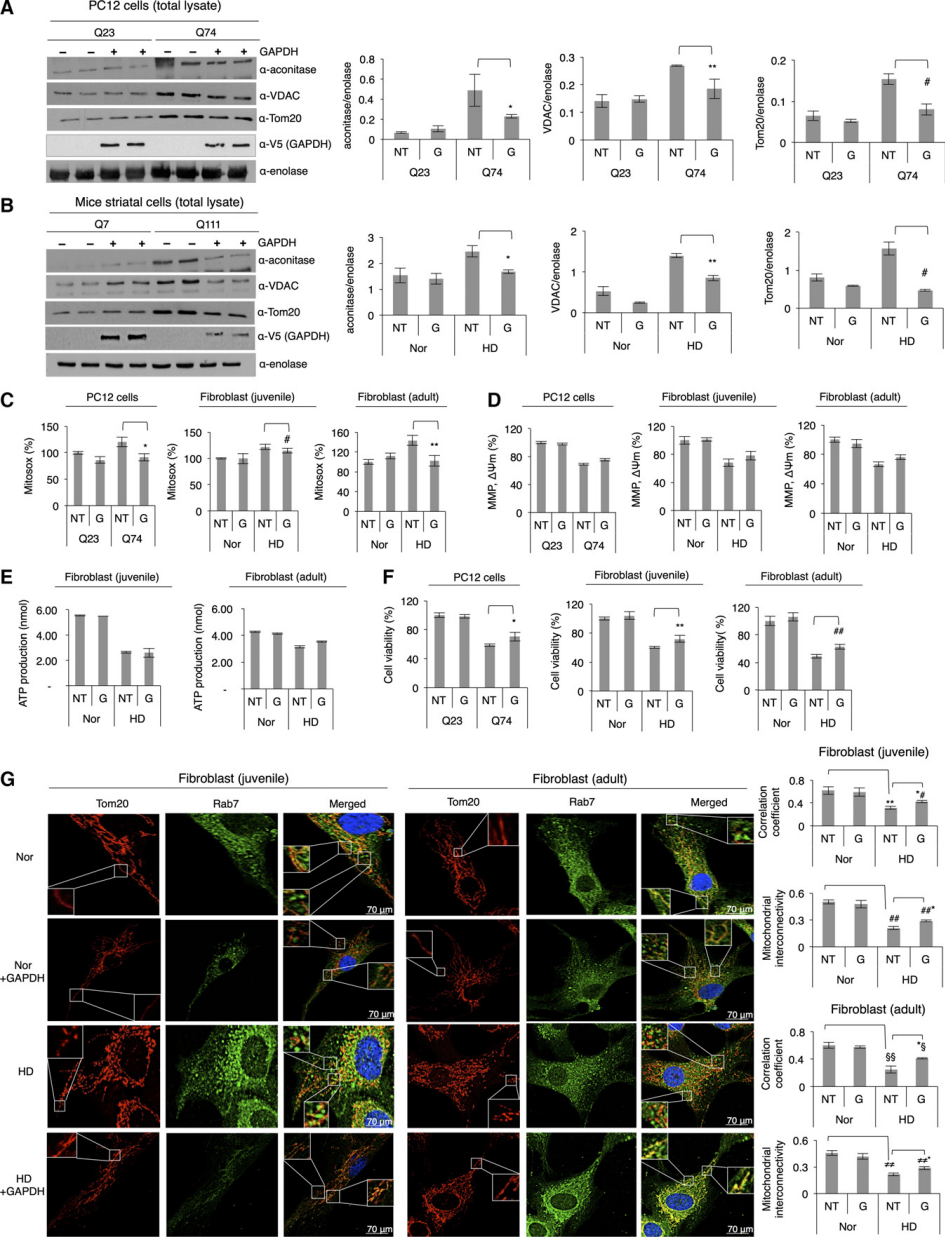
Overexpression of inactive GAPDH rescues polyglutamine-induced blunted mitophagy and recovers mitochondrial function Representative Western blot showing levels of mitochondrial proteins, as indicated by the presence of aconitase, VDAC, and Tom20, in the total lysates of PC12 cells with Q23 or Q74 before (represented as NT: no treatment) and after overexpression of inactive GAPDH (represented as G). *n *=* *3. *P <* *0.035; **P =* *0.001; ^#^*P *=* *0.001.
Representative Western blot showing levels of mitochondrial proteins in the total lysates of mice striatal cells with Q7 or Q111 before (NT) and after overexpression of inactive GAPDH (G). *n *=* *2. *P =* *0.006; **P =* *0.007; ^#^*P *=* *0.001.
Determination of mitochondrial ROS production in different HD models. Results are shown as percent of control. *n *=* *3. *P =* *0.008; ^#^*P *=* *0.054; **P =* *0.03.
Determination of mitochondrial membrane potential using JC-1 in different HD models and control cells (MMP, ΔΨm). The difference in membrane potential between NT (no inactive GAPDH overexpression) and G (inactive GAPDH overexpression) in cells with expanded polyglutamine repeats (PC12 cells with Q74 and HD patient-derived fibroblasts) was not statistically significant. *n *=* *3.
ATP levels in normal and HD patient-derived fibroblasts. The difference in ATP levels between NT (no inactive GAPDH overexpression) and G (inactive GAPDH overexpression) in HD patient-derived fibroblasts was not statistically significant. *n *=* *2.
Cell viability determined by colorimetric assays in different HD models and control cells. *n *=* *3. *P =* *0.02; **P =* *0.02; ^##^*P *=* *0.01.
Immunofluorescence of normal and HD patient-derived fibroblasts, with and without overexpression of inactive GAPDH, stained for late endosome with anti-Rab7 antibody and mitochondria with anti-Tom20 antibody. Images were acquired at 63×  magnification, and brightness and contrast of all images were adjusted by 30%. Correlation coefficient (juvenile): **P =* *0.007; *^#^*P *=* *0.005. Mitochondrial interconnectivity (juvenile): ^##^*P *=* *0.001; ^##^*P =* *0.02. Correlation coefficient (adult): ^§§^*P *=* *0.001; *^§^*P *=* *0.005. Mitochondrial interconnectivity (adult): ^≠≠^*P *=* *0.043; ^≠≠^*P =* *0.015. Data information: The data are presented as mean ± SEM. Statistical significance was assessed by Student’s *t*-test. Source data are available online for this figure.

If increased mitochondrial mass in total cell lysates along with accumulation of mitochondrial proteins in cells is due to inhibition of GAPDH-mediated mitophagy, overexpression of inactive GAPDH should rescue this inhibited mitophagy. To test this hypothesis, inactive GAPDH, in which the catalytically important Cys152 was mutated to Ser, was overexpressed in PC12 cells. Expression of inactive GAPDH appeared to re-induce removal of damaged mitochondria (Fig 5A); while the levels of mitochondrial proteins in cells with Q23, as indicated by the presence of aconitase, VDAC, and Tom20, were unchanged by the overexpression of inactive GAPDH, the levels of mitochondrial proteins in cells with Q74 were markedly reduced, to the levels almost similar to those observed in cells with Q23 (Fig 5A). Overexpression of inactive GAPDH significantly reduced mitochondrial proteins also in mice striatal cells expressing Q111 (Fig 5B) as well as in HD patient-derived fibroblasts (Appendix Fig S4A). No substantial changes in levels of mitochondrial proteins occurred when wild-type active GAPDH was overexpressed in PC12 cells with Q74 (Appendix Fig S4B), supporting the role of inactive GAPDH in clearance of damaged mitochondria. Importantly, mitochondrial ROS levels in PC12 cells expressing Q74 and HD patient-derived fibroblast cells were considerably attenuated by overexpression of inactive GAPDH (Fig 5C), suggesting enhanced elimination of damaged mitochondria. Although significant decreases in mitochondrial membrane potential and ATP production were observed in PC12 cells expressing Q74 and HD patient-derived fibroblasts, overexpression of inactive GAPDH alone did not cause these effects (Fig 5D and E). This is because such measurements depend on the function of normal and healthy mitochondria; thus, removal of damaged mitochondria may not affect those parameters cumulatively in the cell during the experimental time frame. However, overexpression of inactive GAPDH in PC12 cells expressing Q74 and HD patient-derived fibroblasts increased cell survival at least by 10% (Fig 5F). Noticeably, the ROS levels and cell viability in control cells (PC12 with Q23 and normal fibroblasts) were not drastically affected by overexpressed inactive GAPDH, suggesting that inactive GAPDH does not induce mitophagy under normal conditions.

In addition, fibroblasts derived from HD patients were analyzed by immunofluorescence using the late endosome marker, Rab7, and the mitochondrial marker, Tom20. In normal fibroblasts (Nor in Fig 5G), filamentous mitochondria and endosome-originated lysosomal structures were distributed along the cell body; endosomal structures appeared either to be separated from or to lie close to mitochondria, as shown in the expanded panels, corresponding to the results in Fig 3A. Partial colocalization of these organelles may reflect basal autophagy. Overexpression of inactive GAPDH in normal fibroblasts (Appendix Fig S4C) does not affect distribution of those organelles significantly (Nor + GAPDH in Fig 5D), indicating that inactive GAPDH alone does not induce mitochondrial elimination under normal condition. In contrast, consistent with the previous study (Guo *et al*, 2013), more fragmented mitochondria were observed in fibroblasts derived from HD patients (HD in Fig 5D). Notably, the fragmented mitochondria appeared to be less colocalized to the endosome-originated structures and rather accumulated around them in cells. These observations suggest impaired clearance of damaged mitochondria in HD patient-derived fibroblasts. When overexpressed inactive GAPDH in these cells was highly colocalized with mitochondria (Appendix Fig S4C), the fragmented mitochondria recovered filamentous morphology by ∼20% relative to the morphology of control cells (HD + GAPDH in Fig 5D), as indicated by mitochondrial interconnectivity. Also, a greater degree of colocalization between mitochondria and the endosome-originated structures was observed (Fig 5D), as evidenced by a higher (Mander’s) correlation coefficient calculated by ImageJ software with the ICA (Intensity Correlation Analysis) plugin as described (Li *et al*, 2004). Taken together, we confirmed that the impaired GAPDH-driven mitophagy in cells expressing expanded polyglutamine repeats was rescued by overexpression of inactive GAPDH.

### *In vitro* reconstitution of mitophagy using recombinant inactive GAPDH promotes clearance of damaged mitochondria, independently of autophagy

**Figure 6:**
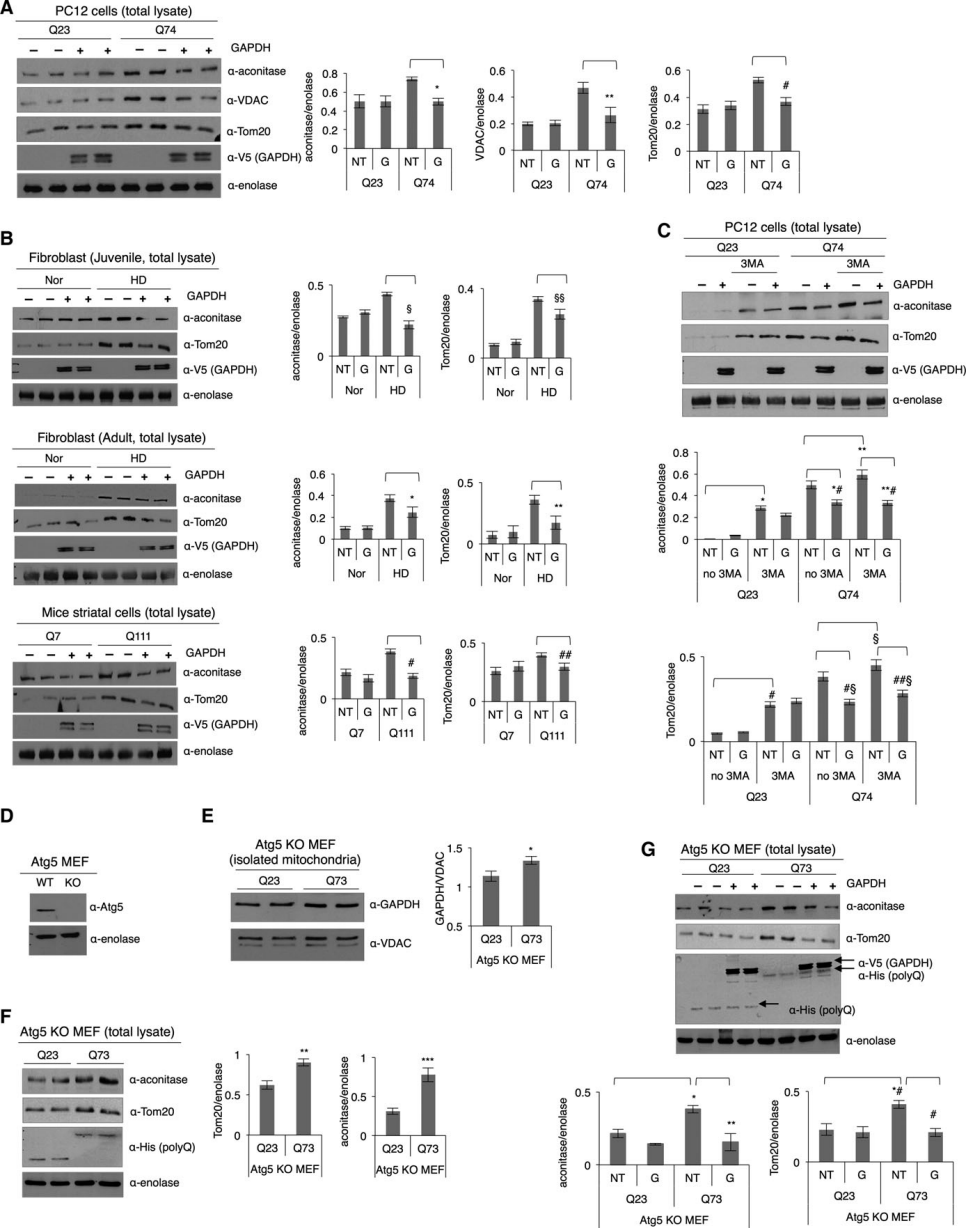
*In vitro* reconstitution of mitophagy using recombinant inactive GAPDH promotes clearance of damaged mitochondria, independently of autophagy Representative Western blot showing levels of mitochondrial proteins, as indicated by the presence of aconitase, VDAC, and Tom20, in the total lysates of PC12 cells with Q23 or Q74 in an *in vitro* reconstituted micro-mitophagic system. NT: no inactive GAPDH treatment; G: inactive GAPDH treatment. *n *=* *3. *P =* *0.0007; **P =* *0.001; ^#^*P *=* *0.008.
Representative Western blots showing levels of mitochondrial proteins (aconitase and Tom20) in the total lysates of fibroblast cells (juvenile and adult) and mice striatal cells with Q7 or Q111 in an *in vitro* reconstituted mitophagic system. *n *=* *2. ^§^*P *=* *0.01; ^§§^*P *=* *0.02; *P =* *0.04; **P =* *0.02; ^#^*P *=* *0.001; ^##^*P *=* *0.024.
Representative Western blot showing levels of mitochondrial proteins (aconitase and Tom20) in the total lysates of PC12 cells. The cells were treated with 1 mM of 3-methyladenine (3MA) for 5 h prior to the micro-mitophagy reconstitution assay. *n *=* *2. *P =* *0.001; **P =* *0.01; *^#^*P *=* *0.003; **^#^*P *=* *0.01; ^#^*P *=* *0.02; ^§^*P *=* *0.049; ^#§^*P *=* *0.004; ^##§^*P *=* *0.007.
Western blot showing the level of Atg5 expression in the Atg5^+/+^ (WT) and Atg5^−/−^ (KO) MEF cells.
Western blot showing levels of GAPDH association with mitochondria in Atg5 KO MEF cells. *n *=* *2. *P =* *0.04.
Western blot showing relative mitochondrial mass in the total lysates of Atg5 KO MEF cells. *n *=* *2. **P =* *0.03; ***P =* *0.02. Expression levels of Q23 or Q73 were detected with anti-His antibody.
Western blot showing relative mitochondrial mass in the total lysates of Atg5 KO MEF cells without and with recombinant inactive GAPDH treatment (represented as NT and G, respectively). *n *=* *2. *P =* *0.02; **P =* *0.03; *^#^*P *=* *0.03; ^#^*P *=* *0.005. Data information: The data are presented as mean ± SEM. Statistical significance was assessed by Student’s *t*-test. Source data are available online for this figure.

An *in vitro* reconstitution assay was used as an additional approach to confirm that GAPDH-driven mitophagy is impaired by expanded polyglutamine repeats. Prior to the reconstitution assay, recombinant GAPDH was incubated first with 0.5 mM H_2_O_2_ for 30 min at 37°C to make it inactive (to mimic GAPDH oxidation under cellular environments), and its decreased enzyme activity was confirmed (Appendix Fig S5A). After a 30-min incubation of isolated organelles containing lysosomes and mitochondria in the total lysates of PC12 cells with inactive GAPDH, the recombinant enzyme associated with the mitochondria (Appendix Fig S5B), which promoted a substantial reduction in mitochondrial mass in the total lysate of PC12 cells with Q74 (Fig 6A), indicating enhanced mitophagy by GAPDH. Similarly, enhanced clearance of damaged mitochondria in the total extracts of HD patient-derived fibroblasts and mice striatal cells with Q111 was observed as well, as evidenced by decreased levels of mitochondrial matrix and outer membrane proteins (aconitase and Tom20), when recombinant inactive GADPH (V5 tagged) was added to organelles in the lysate (Fig 6B).

Finally, to determine whether this elimination pathway occurs independently of autophagy, we incubated PC12 cells with 1 mM of 3-methyladenine (3MA), an inhibitor of autophagosome biogenesis, for 5 h, and lysed cells gently by passing the lysate through a needle to release organelles. The increase in mitochondrial mass, which was observed when Q74 was overexpressed in these cells, was further augmented following the 3MA treatment, as indicated by the increased levels of aconitase and Tom20, suggesting that autophagy is blocked (Fig 6C). Yet, incubating the organelles in the lysate, isolated under such condition, with recombinant inactive GAPDH promoted clearance of damaged mitochondria, which indicates that GAPDH-driven mitophagy occurs independently of a process of autophagy (Fig 6C and Appendix Fig S5C). In support of this observation, when using autophagy-defective Atg5 KO MEFs (Fig 6D) that express Q23 or Q73, we noted an increased level of GAPDH on mitochondria in the Q73-containing cells relative to the Q23 cells (Fig 6E) together with an increase in mitochondrial mass, as indicated by the levels of aconitase and Tom20 (Fig 6F). When the mitophagic machinery was reconstituted with recombinant inactive GAPDH as described earlier, the levels of mitochondrial matrix and outer membrane proteins, aconitase and Tom20, were declined (Fig 6G), suggesting recovery of mitophagy. Together, these findings define a new role of GAPDH as an inducer of autophagy-independent mitophagy and its impairment in HD. These data were confirmed using three different models of HD, and overexpression of inactive GAPDH or reconstitution of the mitophagic machinery with recombinant, inactive GAPDH was sufficient to rescue the inhibited removal of damaged mitochondria associated with HD.

## Discussion

**Figure 7:**
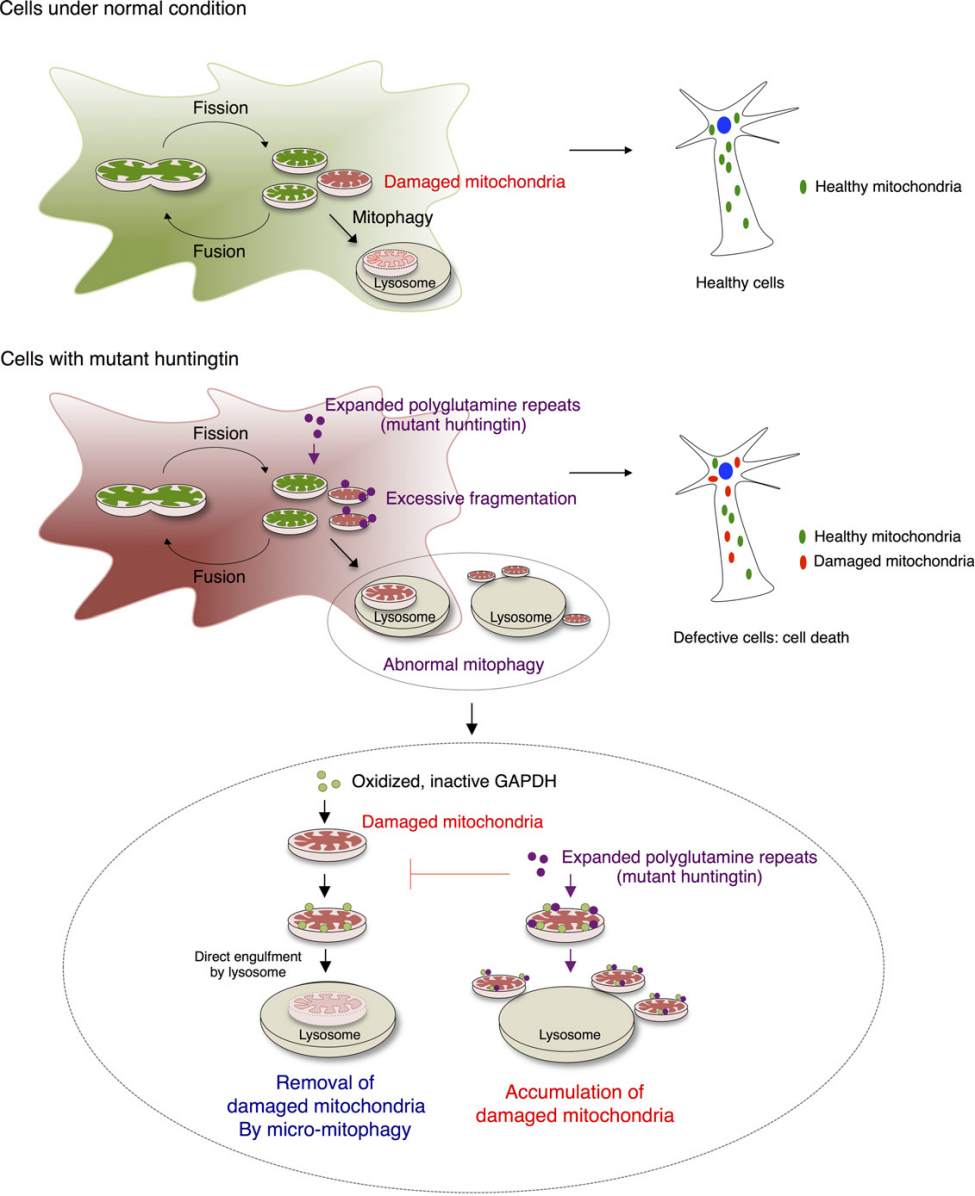
Impaired GAPDH-induced micro-mitophagy contributes to the pathology of HD Schematic illustration of mitochondrial dynamics and GAPDH-mediated micro-mitophagy in HD with expanded polyglutamine repeats.

The processes that lead to HD-associated pathologies are complex. Here, we show that expanded polyglutamine repeats in mutant huntingtin protein impede elimination of damaged mitochondria by mitophagy, thus leading to oxidative stress and cell death. The expanded polyglutamine repeats of mutant huntingtin selectively associated with mitochondrial GAPDH and thus inhibited GAPDH-mediated direct engulfment of damaged mitochondria into the lysosomal system. This, in turn, resulted in accumulation of damaged mitochondria in cells and increased levels of ROS and cell death (Fig 7). Expression of expanded polyglutamine repeats induces oxidative stress and causes a decrease in mitochondrial function by directly interacting with the organelle or mitochondrial proteins (Cui *et al*, 2006; Song *et al*, 2011; Yano *et al*, 2014). Perturbing mitochondrial dynamics, such as increased mitochondrial fragmentation (Cui *et al*, 2006; Reddy *et al*, 2011) and altering a process of mitophagy (Martin *et al*, 2015) (Fig 7) along with impairing mitochondrial trafficking and Ca^2+^ buffering capacity (Choo *et al*, 2004; Chang *et al*, 2006; Orr *et al*, 2008), collectively contribute to the pathology of HD. Such mitochondrial dysfunction is particularly detrimental to neuronal cells, where healthy mitochondria are critical for normal cell functions. Therefore, it is not surprising that mitochondria dysfunctions are implicated in several neurodegenerative diseases including HD.

Increasing lines of evidence demonstrate the importance of mitophagy in neurodegenerative diseases. Regardless of whether mitochondria are randomly or selectively targeted, mitophagy is a crucial process to maintain a pool of functional, healthy mitochondria in cells. The process occurs mainly by either a macro-mitophagic pathway, which is mediated by the engulfment of damaged mitochondria into autophagosomes, or a micro-mitophagic process in which damaged mitochondria are directly taken up via invagination of the lysosomal system (Youle & Narendra, 2011; Li *et al*, 2012). Under pathological conditions of HD (and also other neurodegenerative diseases), mitophagic processes are often impaired, contributing to cellular damages (Cortes & La Spada, 2014; Martin *et al*, 2015). Several steps or phases involved in mitophagy can be disrupted. These include defective autophagosome synthesis (Aguado *et al*, 2010), loss of autophagosomes’ ability to recognize damaged mitochondria or other organelles (Martinez-Vicente *et al*, 2010; Khalil *et al*, 2015), and failure of the autophagosomes to fuse with lysosomes (Martinez-Vicente *et al*, 2010; Wong & Holzbaur, 2014). Furthermore, mitophagy may be impaired because of potential defects of lysosomal functions (Bahr & Bendiske, 2002), leading to deficient elimination by lysosomes. Parkin-mediated mitophagy, initiated by accumulation of PINK1 on damaged mitochondria, is thought to be mainly involved in Parkinson’s disease (Matsuda *et al*, 2010). However, the PINK1/Parkin-mediated pathway is also associated with HD, in a process dependent on expanded polyglutamine repeats (Khalil *et al*, 2015). Along with all these findings, we now demonstrate that removal of damaged mitochondria by a GAPDH-mediated micro-mitophagic pathway is defective in several models of HD. The threshold for clearance of damaged mitochondria via GAPDH was thus raised (Fig 7), suggesting the involvement of defective or blunted GAPDH-driven mitophagy as a mechanism contributing to HD pathology.

It is interesting to note that GAPDH, a ubiquitous enzyme involved in early steps of glycolysis, plays a signaling switch in initiating mitophagy under oxidative stress. GAPDH has been characterized as a ‘moonlighting’ enzyme, performing a number of non-glycolytic functions, including regulations of gene transcription (Zheng *et al*, 2003), microtubule dynamics (Tisdale, 2002), calcium signaling (Patterson *et al*, 2005), and vesicular transport (Bryksin & Laktionov, 2008) and further interacting with a number of key molecules such as glutathione (Puder & Soberman, 1997) and nitric oxide (Hara & Snyder, 2006). Such functional diversity of GAPDH has been closely linked to its oligomerization status, post-translational modifications, and the subcellular localization of the enzyme (Tristan *et al*, 2011). Catalytically active GAPDH primarily exists in cytosol as homotetramer (Seidler, 2013), and the dynamics of GAPDH oligomeric structure, such as dissociation of tetrameric GAPDH into a monomeric form, often reflect diminished enzymatic activity together with acquisition of new roles in diverse subcellular locations, as shown in our study. Most importantly, GAPDH is sensitive to different oxidative post-translational modifications, such as glutathionylation and S-nitrosylation, and Cys residues in its active site are particularly affected by such modifications, leading to inactivation of the enzyme (Colell *et al*, 2009). Nitrosylation of GAPDH by oxidative stressor, nitric oxide, has shown to induce translocation of GAPDH to the nucleus and initiates apoptotic cell death (Hara *et al*, 2005). Given that expanded polyglutamine repeats generate oxidative stress in cells, as shown in our study and by other group (Hands *et al*, 2011), GAPDH can undergo diverse oxidative modifications. Specifically, we confirmed the oxidation of a sulfhydryl group (-SH) of the catalytically important Cys152 of GAPDH to a sulfonic group (-SO_3_), which also contributes to changes in enzyme activity. This modification may further allow GAPDH to translocate to mitochondria, which initiates mitophagy. In support of this, when PC12 cells expressing Q23 or Q74 were treated with a common anti-oxidant, N-acetylcysteine (NAC), GAPDH association with the mitochondria was reduced (Appendix Fig S6A), as NAC should counteract oxidative stress induced by Q74 and in turn, GAPDH is less likely to undergo oxidative modifications. Under these conditions, GAPDH-induced mitophagy may decrease. In the presence of NAC, the decrease in mitochondrial mass observed in the cells expressing Q74 (Appendix Fig S6B) may suggest that complementary autophagic responses may be activated to remove damaged mitochondria or mitochondrial functions are not that damaged. Recent study further showed that NAC has mitochondria-specific anti-oxidant effects, reducing mitochondrial dysfunctions (Xiong *et al*, 1999; Wright *et al*, 2015).

Another attractive hypothesis is that GAPDH in the vicinity of damaged mitochondria is oxidized by the reactive oxygen species (ROS) produced by these mitochondria and in turn binds and tags the damaged mitochondria for elimination. This binding of oxidized GAPDH to the outer membrane of the damaged mitochondria may be mediated by oxidation-dependent exposure of positively charged patch in GAPDH, which enables its binding to the negatively charged head groups of phospholipids (Morero *et al*, 1985; Glaser & Gross, 1995).

The role of mitochondria-associated GAPDH has not been extensively studied, probably because of a low level of GAPDH on mitochondria (< 5%) under normal conditions (Letko *et al*, 1973). However, a recent study demonstrated increased GAPDH levels on mitochondria under stressed conditions, such as treatments by mitochondriotoxic drugs or serum deprivation, inducing apoptotic cell death with an increased level of cytochrome *c* release (Tarze *et al*, 2007). We found that prolonged mitochondrial oxidative stress induced by ischemia–reperfusion causes mitochondrial GAPDH to be phosphorylated by protein kinase C δ (PKCδ) (Yogalingam *et al*, 2013), which blunts the cytoprotective GAPDH-induced mitophagy, thus triggering apoptotic cell death. Mitochondrial GAPDH has also been shown to protect cells from caspase-independent cell death (CICD), by increasing ATP through an elevation in glycolysis and inducing autophagy to clear damaged mitochondria (Colell *et al*, 2007). Collectively, although it is unclear whether in addition to oxidation, other modifications target GAPDH to mitochondria, mitochondrial GAPDH plays diverse, critical roles in response to cellular stress.

GAPDH interacts with other proteins implicated in the pathogenesis of a variety of neurodegenerative diseases. GAPDH binds to the carboxyl terminal of β-amyloid precursor protein (APP) and amyloid beta protein (Aβ) implicated in Alzheimer’s disease (AD) (Schulze *et al*, 1993; Oyama *et al*, 2000) and to other mutant proteins that contain expanded polyglutamine repeats, such as atrophin-1 implicated in dentatorubral-pallidoluysian atrophy (DRPLA) and ataxin-1, which causes spinocerebellar ataxia type 1 (SCA-1) (Burke *et al*, 1996; Koshy *et al*, 1996). A decrease in intracellular glycolytic activity of GAPDH is a phenotype common to all these diseases, providing evidence that abnormal interactions of GAPDH with these proteins mainly affect energy production necessary for neuronal functions (Mazzola & Sirover, 2001, 2002; El Kadmiri *et al*, 2014). Our findings here identify a new role of GAPDH in HD-associated pathology, which leads to accumulation of damaged mitochondria and inhibition of their removal by mitophagy and suggests that it may occur also in other neurodegenerative diseases mentioned above.

It remains unclear why macro-autophagy does not compensate for the impaired micro-autophagy. Whether GAPDH-mediated mitophagy is deployed together with a macro-autophagic pathway to remove damaged mitochondria and whether both pathways are impaired or inhibited with expression of mutant huntingtin with expanded polyglutamine repeats in HD are also unclear. Oxidized GAPDH is also a substrate of chaperone-mediated autophagy (CMA) (Kaushik & Cuervo, 2012), potentially suggesting that GAPDH-mediated mitophagy may also be incorporated into CMA, in a process that remains to be elucidated. Thus, a better understanding of mechanisms associated with elimination of damaged mitochondria will allow us to find an efficacious strategy to prevent or slow down the disease progression. Here, we showed that GAPDH translocates to damaged mitochondria under oxidative stress caused by expression of mutant huntingtin, which is an initial step toward cytoprotective mitophagy. However, mutant huntingtin with expanded polyglutamine repeats selectively associates with GAPDH at the outer mitochondrial membrane, blocking GAPDH-mediated clearance of the damaged mitochondria by lysosomes. Thus, damaged mitochondria accumulate in cells, leading to cell death by apoptosis. Importantly, this mutant huntingtin-induced impairment of mitophagy can be corrected by overexpressing catalytically inactive GAPDH (iGAPDH); iGAPDH enhanced the blunted mitophagy and resulted in improved mitochondrial function and cell survival in cells expressing expanded polyglutamine repeats. These data reflect a critical role of GAPDH-driven mitophagy in HD and suggest that a means to enhance micro-mitophagy provides a potential therapeutic approach to treat HD and maybe other neurodegenerative diseases. Because of the complexity of the disease, a comprehensive treatment, including a selective inhibitor of mitochondrial fission protein (Drp1) and a pharmacological activator of the macro-autophagic pathway (Nixon, 2013) such as mTOR-inhibiting drug, rapamycin, may provide the greatest therapeutic potential.

## Materials and Methods

### Materials

Antibodies used in this study were principally purchased from Santa Cruz Biotechnology (Tom20: SC-11415, enolase: SC-15343, Grp75: SC-1058, V5: SC-58052, cathepsin D: SC-6486, calnexin: SC-11397), Abcam (VDAC: ab14734, cytochrome *c*: ab110325, LAMP1: ab24170, aconitase: ab71440, EGFP: ab111248), Cell Signaling (β-actin: 3700S, Rab7: 9367S), Millipore (huntingtin: MAB 2166, polyglutamine: MAB1574), Advanced ImmunoChemical (GAPDH: 6C5), and Genway Biotech (GADPH-SO_3_: GWB-665A68). Lysosomal enrichment kit (89839) was obtained from Thermo Scientific. MitoTracker Red (M7512), MitoSOX Red (M36008), and JC-1 dye (T3168) were purchased from Invitrogen for immunofluorescence microscopy studies and measurement of mitochondrial function, respectively. Cell Counting Kit-8 (CK04) was purchased from Dojindo to check cell viability. cDNAs encoding polyglutamine repeats Q23 (CH00017) and Q73 (CH00018) were purchased from Coriell Institute (New Jersey, USA).

### Cell culture

A PC12 cell line, where huntingtin exon 1 containing 23 or 74 glutamines fused to enhanced green fluorescent protein (EGFP) is inducibly expressed by doxycycline, was kindly obtained from Dr. David C. Rubinsztein at the University of Cambridge. The cells were cultured in Dulbecco’s modified Eagle medium (DMEM), supplemented with 10% horse serum, 5% fetal bovine serum (FBS), 2 mM l-glutamine, 100 U/ml penicillin, 100 μg/ml streptomycin, 50 μg/ml of G418, and 70 μg/ml hygromycin B, on collagen IV-coated plates. The cells were maintained as previously described (Wyttenbach *et al*, 2001), and expression of Q23 or Q74 was induced for at least 72 h before experiments. HD patient-derived fibroblast cell lines (GM04693 from 33-year-old male patient and GM05539 from 10-year-old male patient) and normal fibroblast cell lines from adult and juvenile as controls were purchased from Coriell Institute and Invitrogen, respectively, and cultured in minimum essential medium supplemented with 15% FBS, 100 U/ml penicillin, and 100 μg/ml streptomycin. Immortalized striatal cell lines, Hdh Q7/7 and Hdh Q111/111 derived from a knock-in transgenic mice containing huntingtin loci with a humanized exon 1 containing Q7 and Q111, were also purchased from Coriell Institute and maintained in DMEM supplemented with 10% FBS, 100 U/ml penicillin, 100 μg/ml streptomycin, and 400 μg/ml G418. All the cells were maintained at 37°C in a humidified incubator with an atmosphere of 5% CO_2_ and 95% air.

### HD transgenic mice

Male R6/2 mice and their wild-type (WT) littermates were purchased from Jackson Laboratories [Bar Harbor, ME; B6CBA-TgN (HD exon1) 62; JAX stock number: 006494] at 5 weeks of age. The R6/2 mice are transgenic for the 5′ end of the human huntingtin gene carrying 100–150 CAG repeats. They had an average of 131 ± 2.3 (mean ± SD) CAG repeats. Mice were maintained under standard 12-h light–dark cycles (lights on at 6 am and off at 6 pm). The life span of those mice was 12–14 weeks in average. Brain samples were taken from the mice at the age of 13 weeks, when they showed severe symptoms of HD (Guo *et al*, 2013). All experiments in animals were in accordance with protocols approved by the Institutional Animal Care and Use Committee of Stanford University and were performed based on the National Institutes of Health Guide for the Care and Use of Laboratory Animals. Sufficient actions were considered for reducing pain or discomfort of subjects during the experiments.

### Mitochondria isolation

Cells were washed with cold PBS and scraped off using mannitol–sucrose (MS) buffer containing 210 mM mannitol, 70 mM sucrose, 5 mM MOPS, 1 mM EDTA, and protease inhibitor cocktail, pH 7.4. The collected cells were passed through a 27-gauge ½-inch needle for lysis, followed by micro-centrifugation at 800 × *g* to pellet nuclei. The post-nuclear supernatant was further micro-centrifuged at 10,000 × *g* for 15 min to collect a mitochondria-enriched fraction as previously described (Yogalingam *et al*, 2013).

### Mitochondria and lysosome enrichment using a density gradient

Mitochondria and lysosomes (from three 100-mm tissue culture dishes) were enriched and isolated by high-speed density gradient centrifugation using a lysosome enrichment kit (Thermo Scientific, 89839) according to the manufacturer’s instructions. Eight fractions were collected from the gradients and used for downstream applications as previously described (Yogalingam *et al*, 2013).

### Fractionation of mitochondrial and lysosomal membranes

Following the protocol of the density gradient, fractions of 1–4 (lysosomal fractions) and 5–8 (mitochondrial fractions) were collected. The pulled fractions were then subjected to a freeze–thaw cycle to break down the organelles, and the lysed organelles were centrifuged at 100,000 × *g* to separate the organelle contents from the organelle membranes. Those fractions were analyzed with respective markers using Western blot.

### Transmission electron microscopy

Intact PC12 cells expressing Q23 or Q74 growing on collagen IV-coated plates and the sections of brain tissues from wild-type mice and HD transgenic mice were fixed with a solution containing 2% (v/v) glutaraldehyde and 4% formaldehyde in 0.1 M sodium cacodylate, pH 7.4, for 15 min at room temperature. The fixed samples were then treated at the Stanford Electron Microscopy Facility for further steps as previously described (Yogalingam *et al*, 2013). Images were acquired using a JEOL1400 transmission electron microscope.

### Morphological analysis of mitochondria in TEM micrographs

The analysis was performed as previously described (Picard *et al*, 2013). Mitochondria in TEM micrographs were individually traced, and shape and size parameters were obtained using ImageJ software. Surface area, representing mitochondrial size, was reported in squared micrometers (μm^2^); form factor, reflecting a branching aspect of mitochondria, was calculated as [(perimeter^2^)/4π surface area)]; roundness, an index of sphericity, was calculated as [4 (surface area)/(π major axis^2^)]; Feret’s diameter measured a longest distance between two points within a mitochondrion; and aspect ratio, reflecting mitochondrial elongation, was calculated as (major axis/minor axis).

### Immunofluorescence

Cells plated on Permanox plastic slides were fixed with 4% formaldehyde in PBS for 10 min at room temperature and blocked with PBS containing 0.1% Triton X-100 and 1% normal goat serum (blocking buffer) for 2 h at room temperature. The cells were incubated with anti-Tom20 antibody (1:250 dilution) and anti-Rab7 antibody (1:100 dilution) in the blocking buffer overnight in a humidified chamber and followed by incubation with Alexa Fluor 546 goat anti-mouse IgG (1:500 dilution) and FITC-conjugated goat anti-rabbit IgG (1:500 dilution). The cells were then washed gently with PBS and counterstained with Hoechst 33342 (1:10,000 dilution, Molecular Probes) to visualize nuclei. The coverslips were mounted with Slowfade antifade reagent (Invitrogen), and images were acquired using an LSM 700 laser scanning confocal microscope (Zeiss) at 63× magnification. Similar procedures were followed to stain cells for GAPDH or V5. In some cases, 5 μM Mitosox reagent was used to stain mitochondria generating superoxide by incubating live cells in HBSS (Hank’s balanced salt solution) for 10 min at 37°C prior to fixation.

### Analysis of immunofluorescence images

The quantification of colocalization between two fluorescence channels was performed by using ImageJ software with the ICA (Intensity Correlation Analysis) plugin as previously described (Li *et al*, 2004) to calculate the Pearson’s correlation coefficients for analysis of images in Figure H and Appendix Figure S4C and the Mander’s correlation coefficient for analysis of images of mitophagy in Figure 5G. Three representative images per condition were analyzed. Parameters of mitochondrial morphology were further quantified with a custom plugin for ImageJ as described (Dagda *et al*, 2009). The average area/perimeter ratio of three independent images was used as an index of mitochondrial interconnectivity.

### Sub-mitochondrial localization with proteinase K

To determine submitochondrial localization of GAPDH and expanded polyglutamine repeats, equal aliquots of mitochondria (20 μg in 30 μl MS buffer) were left untreated or subjected to proteinase K treatment (50 μg/ml) for 30 min at room temperature. To stop protease activity, 5 mM phenylmethylsulfonyl flouride (PMSF) was added and incubated on ice for 5 min. The samples were resolved by SDS–PAGE and analyzed by Western blot with anti-GAPDH, aconitase (mitochondrial matrix protein), mitofusin 1 (MFN1, outer mitochondrial membrane protein), and EGFP (for detection of polyglutamine repeats) antibodies.

### Expression of GAPDH

Overexpression of GAPDH in cells was carried out with Lipofectamine 2000 according to the manufacturer’s instruct-ions (Invitrogen). To express catalytically inactive GAPDH, Cys152 in its active site was mutated to Ser. Transfection was allowed for 24 h, and cells were collected for downstream applications.

### Measurement of mitochondrial ROS production

To determine mitochondrial ROS production, cells were treated with 5 μM Mitosox red mitochondrial superoxide indicator (Invitrogen) for 10 min at 37°C as instructed by manufacturer. The fluorescence was analyzed with excitation/emission at 510/580 nm.

### Measurement of mitochondrial membrane potential

Cells were treated with 5 μM JC-1 dye (Invitrogen) in HBSS (Hank’s balanced salt solution) together with DAPI for 20 min at 37°C, and the fluorescence was analyzed with excitation/emission at 485/590 nm for mitochondrial polarization and at 485/525 nm for mitochondrial depolarization. The mitochondrial depolarization was indicated by a decrease in ratio of polarization to depolarization. The signal was then normalized to nuclei staining.

### Measurement of mitochondrial oxygen consumption

Intact PC12 cells (10^6^ cells/ml) were used to measure mitochondrial O_2_ consumption in culture medium using OROBOROS Oxygraph-2k. ADP (1 mM) and oligomycin (1 μg/ml) were sequentially added to determine state 3 and state 4 respiratory rate to calculate RCR (respiratory control ratio), a parameter demonstrating the tightness of the coupling between mitochondria respiration and phosphorylation (Guo *et al*, 2013). Following oligomycin addition, FCCP (1 μM) and antimycin A (2.5 μM) were sequentially injected.

### Measurement of ATP level

The cellular ATP concentration was measured using an ATP bioluminescence assay (Invitrogen, NY) for quantitative determination of ATP with recombinant firefly luciferase and its substrate D-luciferin. The assay is based on luciferase’s absolute requirement for ATP in producing light (emission at 560 nm at pH 7.8). Cells were washed in PBS (pH 7.0) and lysed in 100 μl of 1% trichloroacetic acid. About 2.5 μl of the lysate was used to determine the total ATP level using a luminometer according to the manufacturer’s instructions.

### Cell viability assay

Cell viability was measured using a Cell Counting kit-8 (CCK-8, Dojindo), utilizing WST-8 [2-(2-methoxy-4-nitrophenyl)-3-(4-nitrophenyl)-5-(2,4-disulfophenyl)-2H-tetrazolium salt], according to the manufacturer’s instructions. Some cells were subjected to serum starvation for 48 h before measurement. Cells in 100 μl of medium per well were treated with 10 μl of CCK-8 solution and incubated up to 4 h. The absorbance was read at 450 nm.

### Cross-linking co-immunoprecipitation

PC12 cells with Q23 or Q74 were washed gently with PBS buffer, and proteins were cross-linked by incubating the cells in PBS with 1% formaldehyde for 20 min at room temperature. The cross-linking reaction was terminated by incubating cells in PBS containing 100 mM glycine (pH 3.0). The cells were then washed with PBS and lysed in 0.5 ml PBS buffer containing 1% Triton X-100 and protease inhibitor. After determination of protein concentration, 300 μg of cell lysate was immunoprecipitated with anti-GAPDH antibody overnight at 4°C. The immunoprecipitates were then washed, resuspended in the sample buffer, and boiled at 95°C to reverse cross-linking (Klockenbusch & Kast, 2010).

### Reconstitution assay

Cells were gently washed with cold PBS and scraped off from the plates using mannitol–sucrose buffer. Cells were then passed through a syringe needle for lysis. Organelles in the total lysates containing both mitochondria and lysosomes were incubated with recombinant inactive GAPDH for 30 min at 37°C to induce mitophagy *in vitro*. To monitor mitochondrial degradation, samples were analyzed by Western blot with mitochondrial marker antibodies.

### Statistical analysis

All data are presented as mean ± standard error of the mean (SEM). Statistical differences between mean values for the experiments were evaluated by Student’s *t*-test using GraphPad Prism software. *P*-values and number of experiments are denoted within the figure legends, and *P *<* *0.05 was considered significant. Analysis of immunofluorescence images and electron micrographs was carried out by an observer blinded to the experimental conditions.

## Supporting Information

Appendix

Review Process File
