# Supplementary material for: Impaired GAPDH-induced mitophagy contributes to the pathology of Huntington’s disease
Source: EMBO Mol Med. 2015 Aug 12;7(10):1307–26. doi: 10.15252/emmm.201505256 (PMC4604685; doi:10.15252/emmm.201505256)
Supplement: Supplementary file 1 [file emmm0007-1307-sd1.pdf]

Appendix

Impaired GAPDH-induced mitophagy contributes to the pathology of Huntington's disease

Authors
Sunhee Hwang, Marie-Hélène Disatnik, Daria Mochly-Rosen

Affiliation
Department of Chemical and Systems Biology, Stanford University School of Medicine, Stanford, CA 94305-5174, USA

Contact information
mochly@stanford.edu

Running title (50 characters including spaces): Impaired GAPDH-induced mitophagy in HD

Table of Contents:

Appendix Figures
Appendix Figure legends
Appendix Supplementary Experimental Procedures

Figure S1

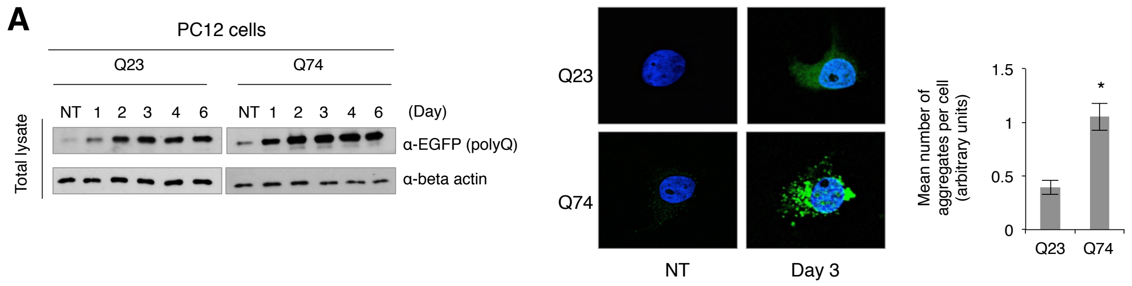
A
PC12 cells
Q23
Q74
NT 1 2 3 4 6
NT 1 2 3 4 6
(Day)
Total lysate
α-EGFP (polyQ)
α-beta actin
Q23
Q74
NT
Day 3
Mean number of aggregates per cell (arbitrary units)
1.5
1
0.5
0
*
Q23
Q74

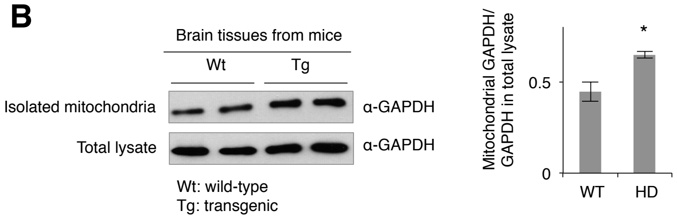
B
Brain tissues from mice
Wt
Tg
Isolated mitochondria
Total lysate
α-GAPDH
α-GAPDH
Wt: wild-type
Tg: transgenic
Mitochondrial GAPDH/ GAPDH in total lysate
0.5
0
*
WT
HD

Figure S2

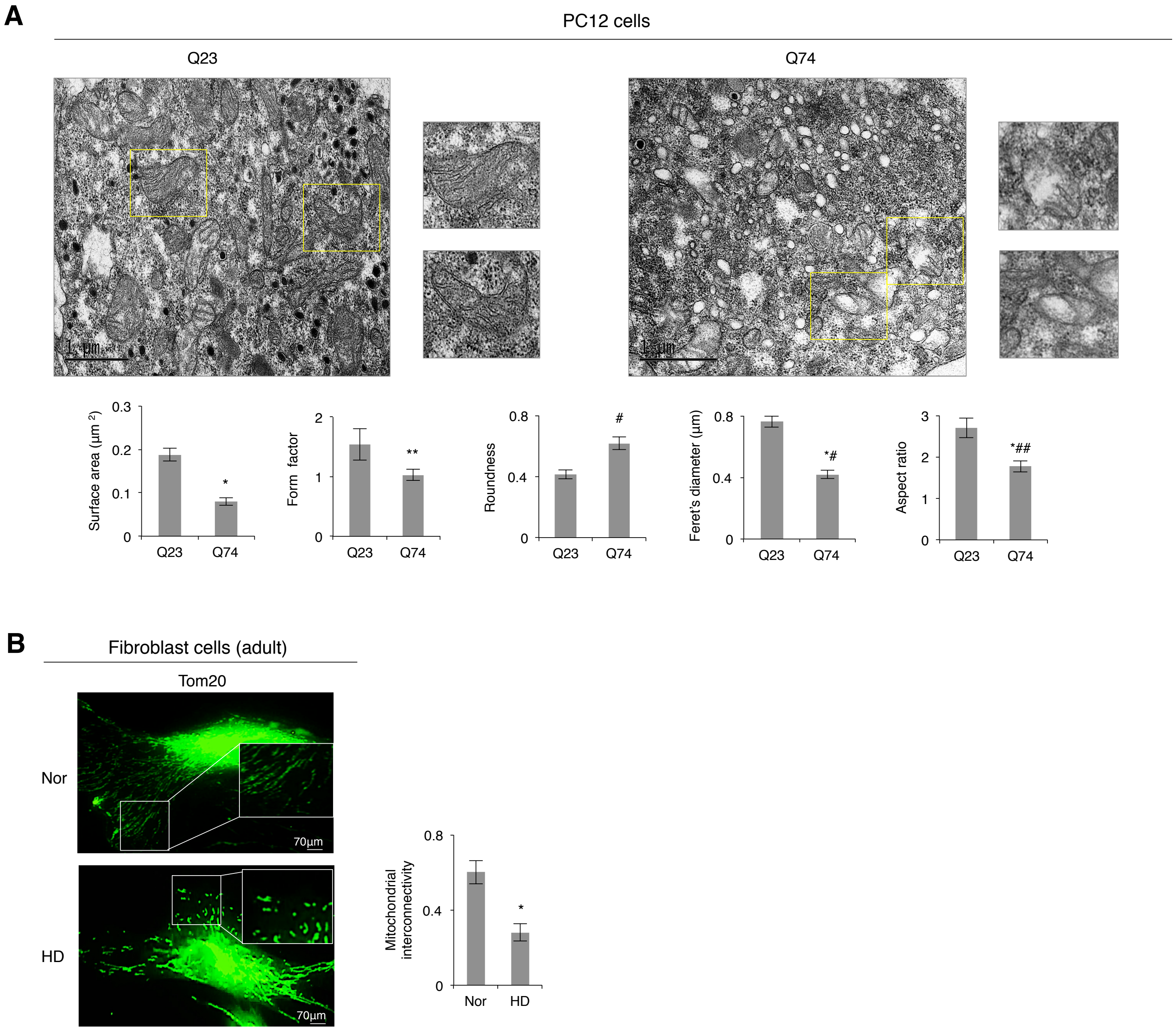
A
PC12 cells
Q23
Q74
1 μm
1 μm
Surface area (μm 2)
0.3
0.2
0.1
0
Q23
Q74
*
Form factor
2
1
0
**
Roundness
0.8
0.4
#
Feret's diameter (μm)
*#
Aspect ratio
3
*##
B
Fibroblast cells (adult)
Tom20
Nor
HD
70μm
70μm
Mitochondrial interconnectivity
0.8
0.4
0
*
Nor
HD

Figure S3

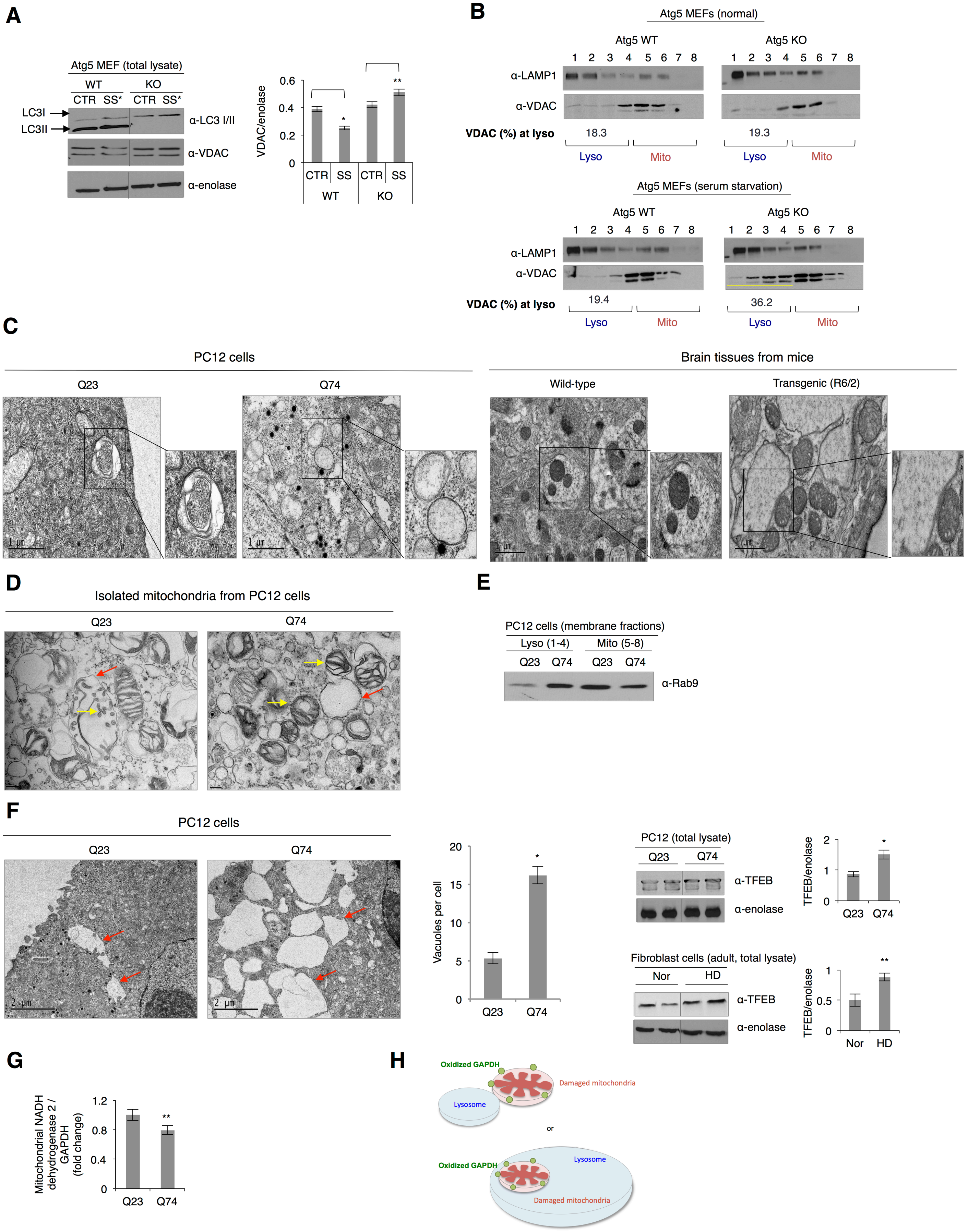
A
Atg5 MEF (total lysate)
WT
KO
CTR
SS*
LC3I
LC3II
α-LC3 I/II
α-VDAC
α-enolase
VDAC/enolase
0.6
0.4
0.2
0
*
**
CTR
SS
WT
KO
B
Atg5 MEFs (normal)
Atg5 WT
Atg5 KO
1 2 3 4 5 6 7 8
α-LAMP1
α-VDAC
VDAC (%) at lyso
18.3
19.3
Lyso
Mito
Atg5 MEFs (serum starvation)
19.4
36.2
C
PC12 cells
Q23
Q74
Brain tissues from mice
Wild-type
Transgenic (R6/2)
D
Isolated mitochondria from PC12 cells
E
PC12 cells (membrane fractions)
Lyso (1-4)
Mito (5-8)
α-Rab9
F
PC12 cells
Vacuoles per cell
20
15
10
5
0
PC12 (total lysate)
α-TFEB
TFEB/enolase
2
1
Fibroblast cells (adult, total lysate)
Nor
HD
0.5
G
Mitochondrial NADH dehydrogenase 2 / GAPDH (fold change)
1.2
0.8
0.4
H
Oxidized GAPDH
Damaged mitochondria
Lysosome
or

Figure S4

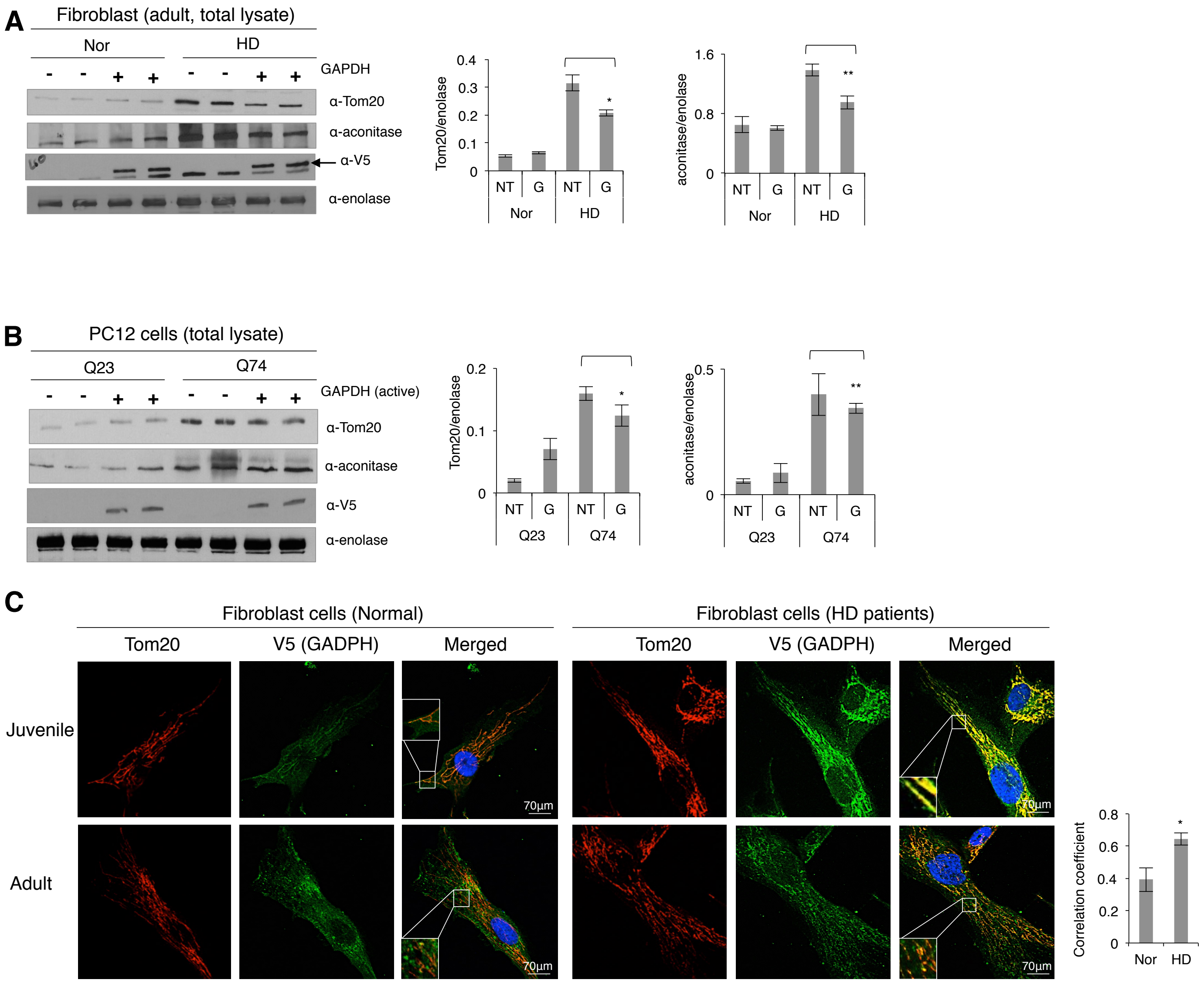

A
Fibroblast (adult, total lysate)
Nor
HD
GAPDH
- - + + - - + +
α-Tom20
α-aconitase
α-V5
α-enolase
Tom20/enolase
0.4
0.3
0.2
0.1
0
NT G NT G
Nor HD
aconitase/enolase
1.6
0.8
0
NT G NT G
Nor HD
B
PC12 cells (total lysate)
Q23
Q74
GAPDH (active)
- - + + - - + +
α-Tom20
α-aconitase
α-V5
α-enolase
Tom20/enolase
0.2
0.1
0
NT G NT G
Q23 Q74
aconitase/enolase
0.5
0
NT G NT G
Q23 Q74
C
Fibroblast cells (Normal)
Fibroblast cells (HD patients)
Tom20
V5 (GADPH)
Merged
Juvenile
Adult
70μm
Correlation coefficient
0.8
0.6
0.4
0.2
0
Nor HD

Figure S5

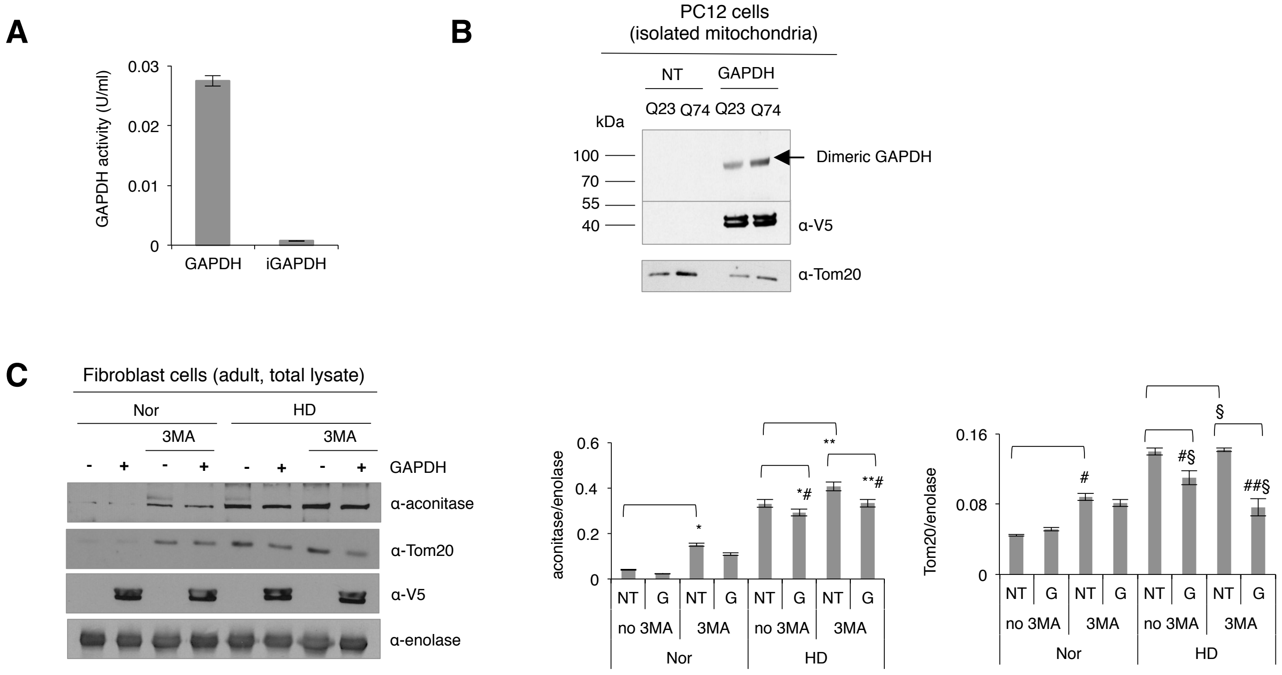
A
GAPDH activity (U/ml)
0.03
0.02
0.01
0
GAPDH
iGAPDH
B
PC12 cells
(isolated mitochondria)
NT
GAPDH
Q23 Q74 Q23 Q74
kDa
100
70
55
40
Dimeric GAPDH
α-V5
α-Tom20
C
Fibroblast cells (adult, total lysate)
Nor
HD
3MA
3MA
- + - + - + - +
GAPDH
α-aconitase
α-Tom20
α-V5
α-enolase
aconitase/enolase
0.6
0.4
0.2
0
NT G NT G NT G NT G
no 3MA
3MA
no 3MA
3MA
Nor
HD
Tom20/enolase
0.16
0.08
0
#
#§
##§
§

Figure S6

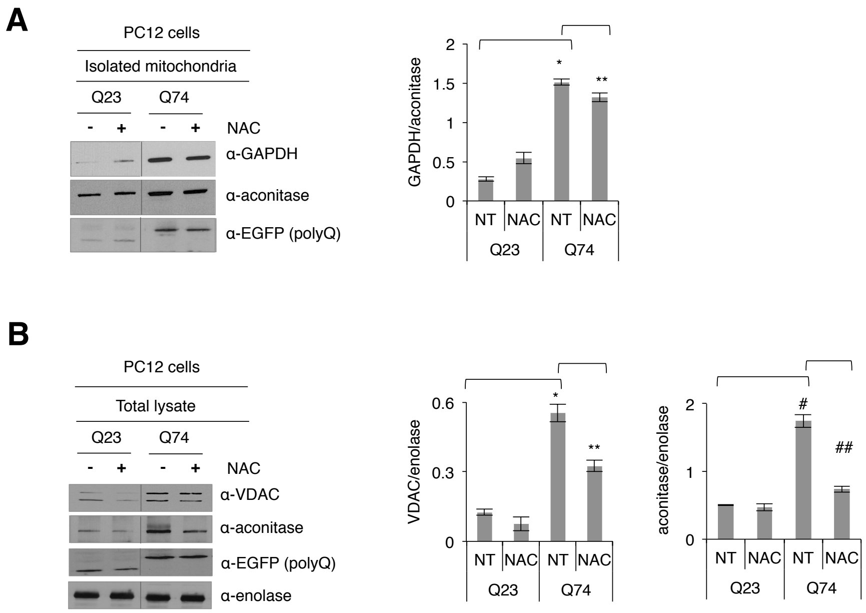

A
PC12 cells
Isolated mitochondria
Q23
Q74
NAC
α-GAPDH
α-aconitase
α-EGFP (polyQ)
GAPDH/aconitase
NT
NAC
B
PC12 cells
Total lysate
α-VDAC
α-aconitase
α-EGFP (polyQ)
α-enolase
VDAC/enolase
aconitase/enolase

Appendix Figure Legends

Appendix Figure S1. **GAPDH selectively associates with mitochondria in cells expressing expanded polyglutamine repeats.** A. (Left) Western blot showing expression levels of polyglutamine repeats in PC12 cells induced by 1μg/ml of doxycycline. (Right) Representative fluorescence images showing expression of Q23 or Q74 in a single cell of PC12, represented by EGFP conjugated to polyglutamine repeats. Mean number of aggregates per cell was calculated by using ImageJ software. NT: Non-treated by doxycycline. $^{*}P$=0.0005. B. Levels of GAPDH in isolated mitochondrial fractions and total extracts of brain tissues from wild-type (Wt) and HD transgenic (Tg) mice. n=2. $^{*}P$=0.034. The data are presented as mean ± SEM.

Appendix Figure S2. **Expanded polyglutamine repeats cause structural abnormalities of mitochondria**. A. Electron micrographs showing tubular-shaped mitochondria in cells with Q23 and more fragmented and smaller mitochondria in cells with Q74. Indicated portions of each image are expanded to illustrate the mitochondrial morphology. Morphological analysis of four representative images per condition was performed using ImageJ. Surface area reflects mitochondrial size; form factor reflects a branching aspect of mitochondria; roundness indicates an index of sphericity; Feret's diameter measures a longest distance between two points within a mitochondrion; and aspect ratio reflects mitochondrial elongation. $^{*}P$, $^{**}P$, $^{\#}P$, $^{*\#}P$, $^{*\#\#}P$=0.0001. B. Immunofluorescence of normal (Nor) and HD patient-derived fibroblasts (adult) stained for mitochondria with anti-Tom20 antibody. Mitochondrial interconnectivity was calculated as the mean area/perimeter ratio. Expanded portions indicate filamentous and fragmented mitochondrial structures, respectively. Images were acquired at × 63 magnification, and brightness and contrast of images were adjusted by 50%. n=3. $^{*}P$=0.002.

The data are presented as mean ± SEM.

Appendix Figure S3. **Accumulation of damaged mitochondria by mitophagy in cells containing expanded polyglutamine repeats.** A. Representative Western blot showing LC3I and LC3II levels in the total lysate of Atg5 WT and KO MEFs. CTR: control; SS*: serum starvation (24 hours). Levels of a mitochondrial marker protein, VDAC, were quantified. *n*=3. *P=0.012; **P=0.043. B. Representative Western blots showing separation of lysosome- and mitochondria-enriched fractions isolated from Atg5 WT and KO MEFs by a density gradient. Eight fractions were collected from top (#1) to the bottom (#8). The amount of mitochondria in the lysosomal fractions was calculated as a percentage of VDAC present in the entire gradient. The yellow bar indicates accumulated mitochondria in the lysosomal fractions. C. Electron micrographs showing double-membraned autophagosomes (as indicated in a black rectangular box) for autophagy in PC12 cells and brain tissues from wild-type and HD transgenic mice. D. Electron micrographs of isolated mitochondria from PC12 cells with Q23 or Q74 showing accumulated mitochondria around lysosomes (yellow arrows: mitochondria; red arrows: lysosomal vacuoles). E. Western blot showing the level of late endosome in membrane fractions isolated from PC12 cells, as indicated by anti-Rab7 antibody. F. (left) Electron micrographs of PC12 cells with Q23 or Q74 emphasizing the number of vacuoles observed. The number of vacuoles per cell was quantified. 6 cell profiles per condition. *P=0.0001. (right) Western blot showing levels of TFEB (marker for lysosomal biogenesis) in the total lysates of PC12 cells with Q23 or Q74 and normal and HD patient-derived fibroblasts (adult). *n*=2. *P=0.032; **P<0.044. G. Fold change in expression level of mitochondrial NADH dehydrogenase 2 (mt-ND2) (normalized to GAPDH). *n*=2. *P=0.005. H. Illustration describing a selective association of oxidized, inactive GAPDH (represented by green circles) with damaged mitochondria in cells expressing expanded polyglutamine repeats. GAPDH-bound damaged mitochondria may be accumulated around the lysosomal system and/or engulfed and internalized directly into them without degradation.

The data are presented as mean ± SEM.

Appendix Figure S4. **Overexpression of inactive GAPDH rescues polyglutamine-induced blunted mitophagy and recovers mitochondrial function.** A. Western blot showing levels of mitochondrial mass, as indicated by the presence of Tom20 and aconitase, in the total lysates of fibroblast cells (adult) with and without overexpression of inactive GAPDH. NT: no inactive GAPDH expression; G: inactive GAPDH overexpression. n=2. $^{*}P$=0.05; $^{**}P$<0.032. B. Western blot showing levels of mitochondrial mass in the total lysates of PC12 cells with Q23 or Q74 with and without overexpression of active GAPDH. The difference in the levels of mitochondrial mass between NT (no active GAPDH overexpression) and G (active GAPDH overexpression) in PC12 cells with Q74 was not statistically significant. n=2. $^{*}P$=0.48; $^{**}P$=0.19. C. Immunofluorescence of normal and HD patient-derived fibroblast cells stained for overexpressed inactive GAPDH with anti-V5 antibody and for mitochondria with anti-Tom20 antibody. Images were acquired at × 63 magnification, and brightness and contrast of images were adjusted by 30%. Three independent images per condition were quantified to calculate the correlation coefficient for colocalization between mitochondria and V5 (overexpressed inactive GAPDH). $^{*}P$= 0.001.

The data are presented as mean ± SEM.

Appendix Figure S5. ***In vitro* reconstitution of mitophagy using recombinant inactive GAPDH promotes clearance of damaged mitochondria, independently of autophagy.** A. Enzymatic activity of recombinant purified GAPDH and inactivated GAPDH (iGAPDH) by H_2O_2. B. Western blot showing association of recombinant enzyme (V5-tagged GAPDH) with isolated mitochondrial fractions and subsequent changes in mitochondrial mass, as indicated by the presence of Tom20. NT: no GAPDH treatment. C. Western blot showing levels of mitochondrial mass in the total lysates of normal (Nor) and HD patient (adult)-derived fibroblast cells. The cells were subjected to the 3-

methyladenine (3MA, 1mM) treatment for 5 hours prior to the reconstitution assay. Total lysates were incubated with inactive GAPDH and analyzed by Western blotting with anti-aconitase and Tom20 antibodies. NT: no inactive GAPDH expression; G: inactive GAPDH overexpression. *n*=2. $^{*}P$=0.038; $^{**}P$=0.047; $^{*\#}P$=0.047; $^{**\#}P$=0.019; $^{\#}P$=0.01; $^{\S}P$=0.08; $^{\#\S}P$=0.037; $^{\#\#\S}P$=0.01.

The data are presented as mean ± SEM.

Appendix Figure S6. **Reduced mitochondrial GAPDH and declined mitochondrial mass in the presence of anti-oxidant, NAC**. A. Western blot showing levels of GAPDH in mitochondria-enriched fractions from PC12 cells with Q23 or Q74 in the absence (denoted as NT: no treatment) or presence of anti-oxidant, N-acetylcysteine (NAC). The cells were treated with 4mM of NAC while polyglutamine expression was being induced by doxycycline. *n*=2. $^{*}P$=0.001; $^{**}P$<0.048. B. Western blot showing levels of mitochondrial mass (as indicated by VDAC and aconitase) in the total lysates of PC12 cells with Q23 or Q74 in the absence (denoted as NT: no treatment) or presence of NAC. *n*=2. $^{*}P$=0.004; $^{**}P$=0.01; $^{\#}P$=0.003;$^{\#\#}P$=0.001.

The data are presented as mean ± SEM.

Appendix Supplementary Experimental Procedures

Western Blot Analysis

Protein concentration was determined by Bradford assay using a Bio-rad protein assay dye reagent (Bio-rad). The protein samples were resuspended in SDS-PAGE sample buffer, boiled for 10 min, and elecrophoresed, and transferred onto nitrocellulose membranes. The transferred proteins were blocked by either 5% milk or BSA solution in Phosphate-Buffered Saline Tween-20 (PBST), incubated with primary antibody overnight at 4°C and the appropriate secondary-HRP conjugated antibody for 2 hours at room temperature. Immunoreactions were visualized and detected. Quantification of signal intensities on Western blots was done using ImageJ software.

Immunoprecipitation

Samples were incubated with the indicated primary antibody in buffer containing 50mM Tris-HCl, pH 7.4, 150mM NaCl, 1% Triton X-100, and protease inhibitor overnight at 4°C with a gentle agitation. Protein A/G beads were then added, and the mixture was incubated for 2 additional hours at 4°C. The mixture was centrifuged for 3 min at 2,000 rpm and the immunoprecipitates were washed three times with buffer, analyzed by SDS-PAGE, and followed by Western blot.

Preparation of recombinant GAPDH

GAPDH cDNA was cloned into the pET16b vector. The resulting plasmid was transformed into chemically competent BL21 (DE3) *E. coli* strain, which was grown on a LB agar plate supplemented with ampicillin overnight at 37°C. Streaks of the colonies on the plate were used to grow 1L of bacteria in terrific broth medium with shaking (200rpm) at 37°C. When the optical density (OD_{600}) reached around 0.6, 0.5mM IPTG was added to induce GAPDH expression. After 4 hour-post induction, the bacteria were collected by centrifugation. The pellet was then lysed by sonication in buffer containing 50mM Tris, pH 7.4, 300mM NaCl, 5% glycerol, 0.4mM PMSF, and 50mg lysozyme.

GAPDH was purified by Ni (II)-affinity chromatography. The final concentration GAPDH was determined by Bradford assay, and the protein was stored at -80°C with 40% glycerol. To make the enzyme inactive (to mimic oxidized GAPDH), 100ug of recombinant GAPDH was incubated with 0.5mM H_2O_2 for 30 min at 37°C and decreased enzymatic activity was confirmed as described below.

Measurement of GAPDH activity

The enzymatic activity of GAPDH was determined using a KDalert GAPDH assay kit as instructed by manufacturer (Invitrogen).

RNA extraction and reverse transcriptase-qPCR reaction

RNA from PC12 cells (one 100mm plate) was isolated in duplicate according to the manufacture protocol using RNAqueous kit (AM1912, Ambion) in 700μl lysis solution per plate. RNA was eluted in 50 μl elution solution, and then 1 mg RNA template was used to synthesize first strand cDNA using PrimeScript RTase-derived reverse transcriptase (6110A, Takara Bio Inc) with Oligo dT primer following the manufacture protocol. 1.5 ng cDNA was used to amplify mitochondrial gene, rat mt-ND2 (mitochondrial NADH dehydrogenase 2), and housekeeping gene, rat GAPDH, by qPCR using fast SYBR green master mix (Applied Biosystems) and 100 nM of a mixture of forward and reverse respective primers in 20 μl total volume reaction (forward primer for mt-ND2: 5'-AGAACCCATACGCCCCCTAACCACC-3'; reverse primer for mt-ND2: 5'-GGGGGTGAGGTATTGGTAAGGGGATTC-3'. Forward primer for GAPDH: 5'-CTCAGTTGCTGAGGAGTCCC-3'; reverse primer for GAPDH: 5'-ATTCGAGAGAAGGGAGGGCT-3'). PCR steps were as follows: Step 1, 95°C for 20 sec; Step 2, 95°C for 3 sec; Step 3, 60°C for 30 sec; Step 4, go to step 3 (40 repeats); Step 5-7 melting curve, 95°C for 15 sec, 60°C for 1 min, 95°C for 15 sec; Step 8, keep at 4°C indefinitely. Quantification was

carried out using analysis of the difference in threshold amplification between mitochondrial DNA and housekeeping DNA (ΔΔC(t) method).
