# Supplementary material for: Impaired GAPDH-induced mitophagy contributes to the pathology of Huntington’s disease
Source: EMBO Mol Med. 2015 Aug 12;7(10):1307–26. doi: 10.15252/emmm.201505256 (PMC4604685; doi:10.15252/emmm.201505256)
Supplement: Supplementary file 2 [file emmm0007-1307-sd2.pdf]

EMBO Molecular Medicine

Impaired GAPDH-induced mitophagy contributes to the pathology of Huntington's disease

Sunhee Hwang, Marie-Helene Disatnik and Daria Mochly-Rosen

Corresponding author: Daria Mochly-Rosen, Stanford University

Review timeline:

Submission date:	16 March 2015
Accepted:	21 July 2015

Transaction Report:

No Peer Review Process File is available with this article, as the authors have chosen not to make the review process public in this case.

Editor: Céline Carret

© EMBO
