# Supplementary figures and images for: Impaired GAPDH-induced mitophagy contributes to the pathology of Huntington’s disease
Source: EMBO Mol Med. 2015 Aug 12;7(10):1307–26. doi: 10.15252/emmm.201505256 (PMC4604685; doi:10.15252/emmm.201505256)

Figure 1

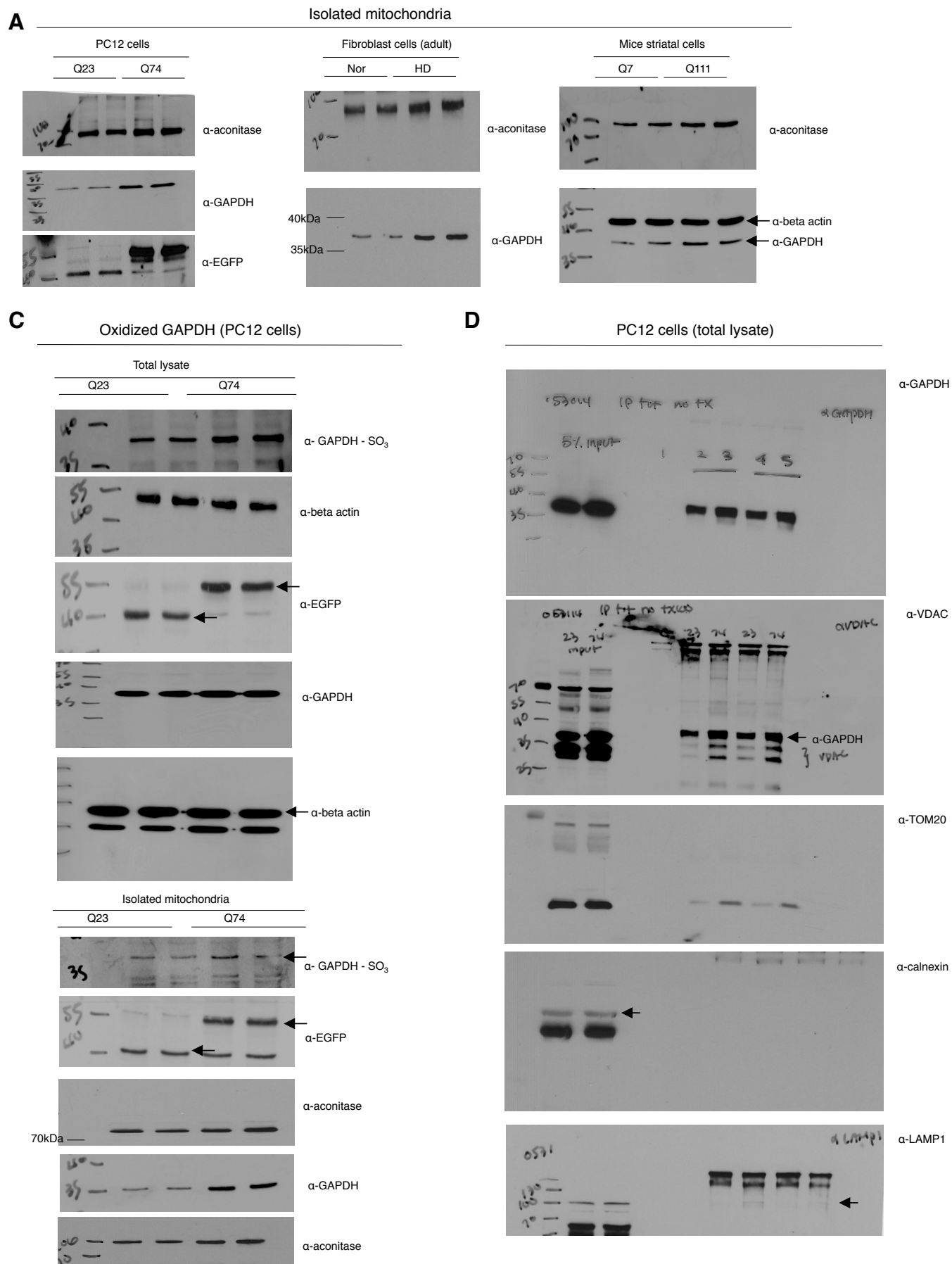

Supplement: Supplementary file 3 [file emmm0007-1307-sd3.pdf]

Figure 2

F

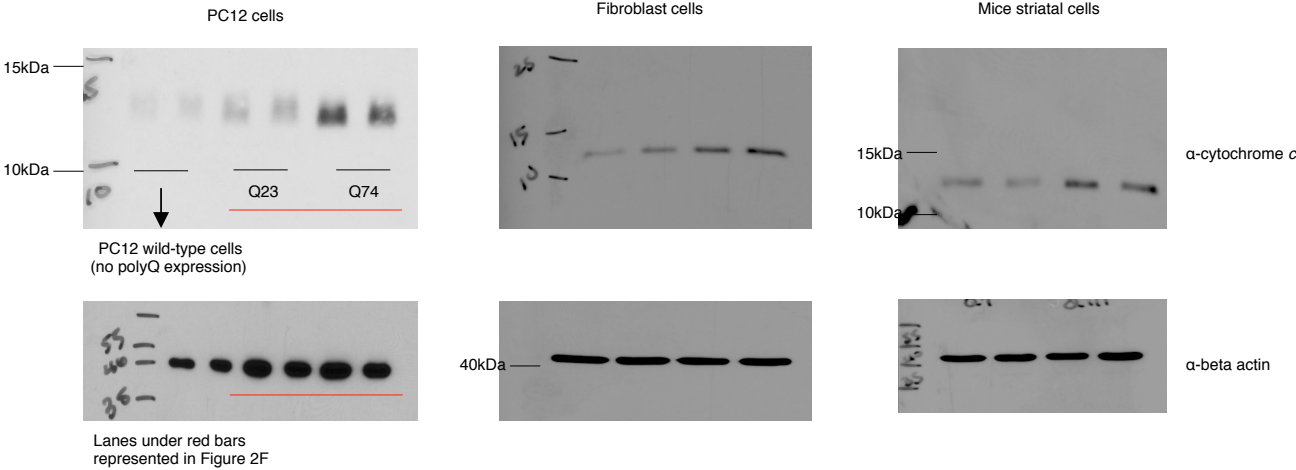

H

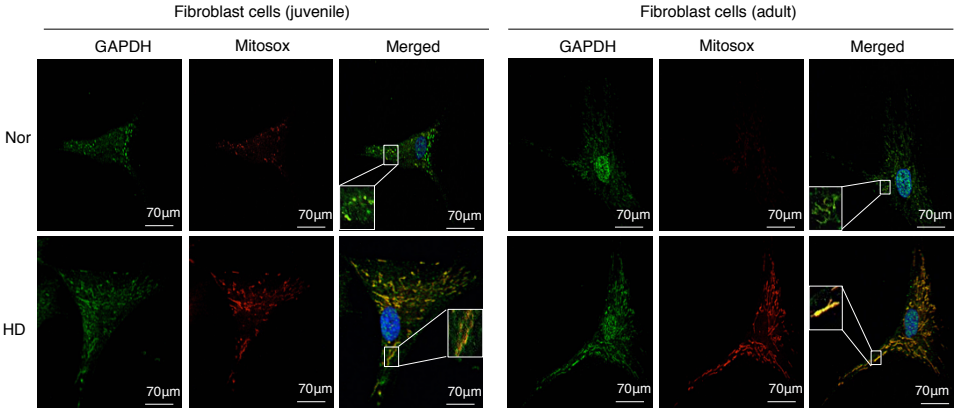

Supplement: Supplementary file 4 [file emmm0007-1307-sd4.pdf]

Figure 3

A

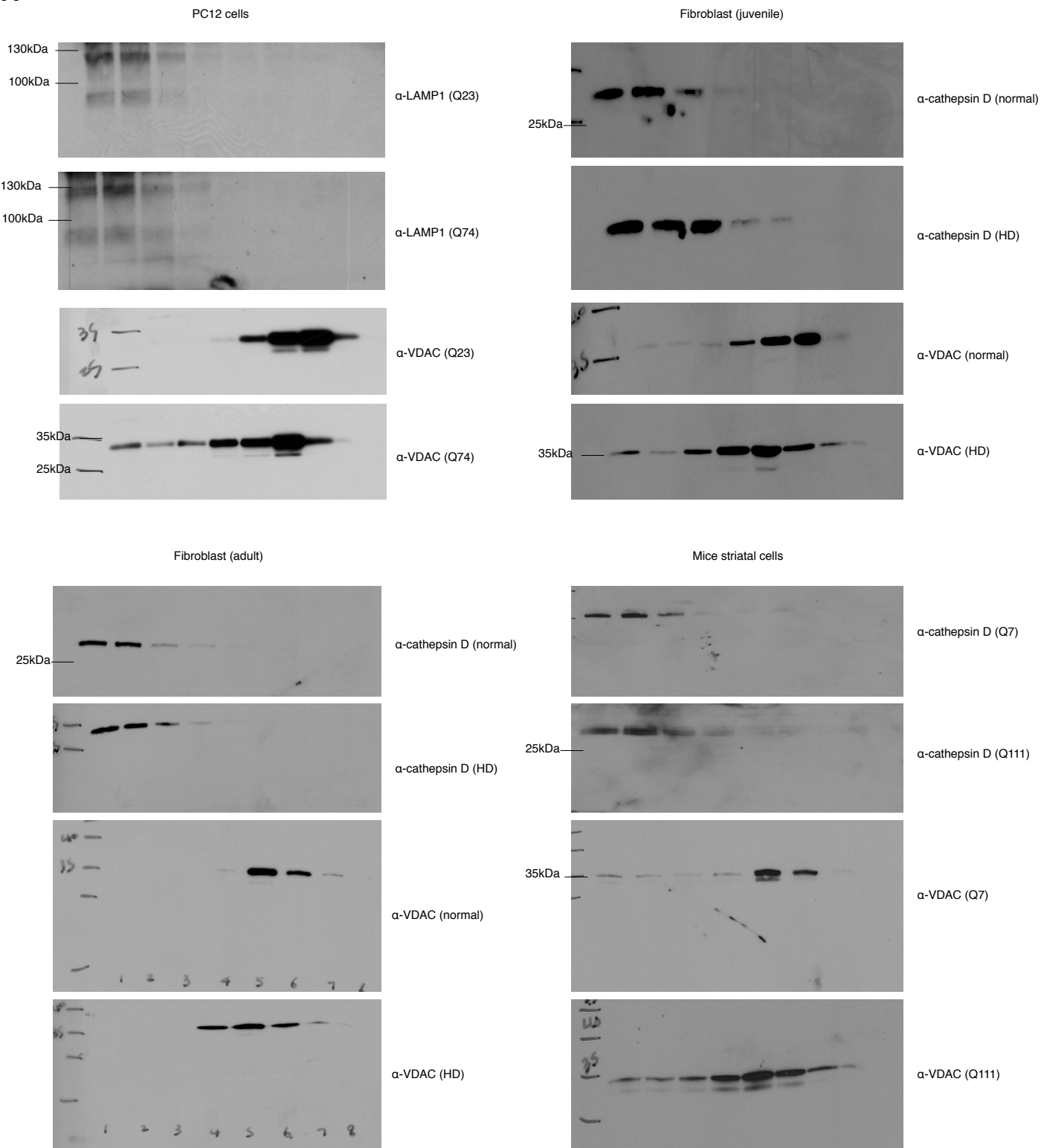

Figure 3

D

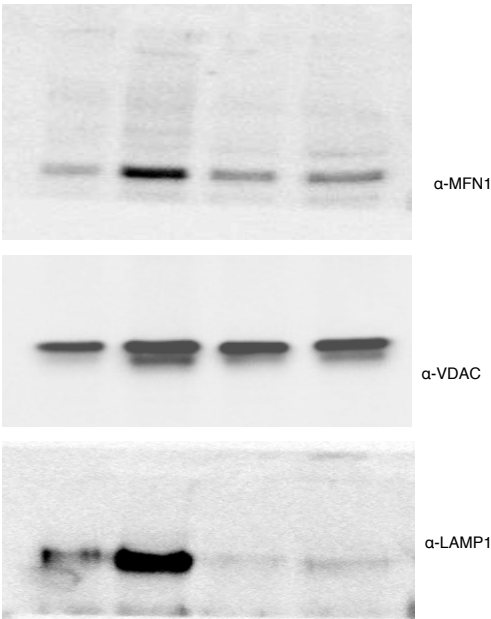

E

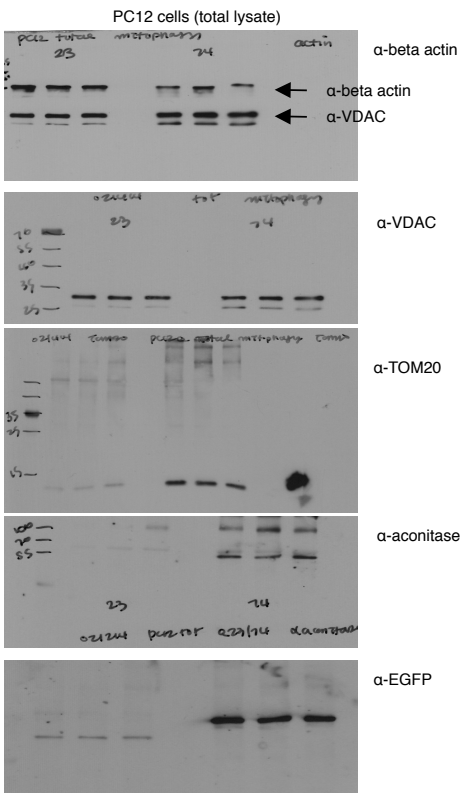

F

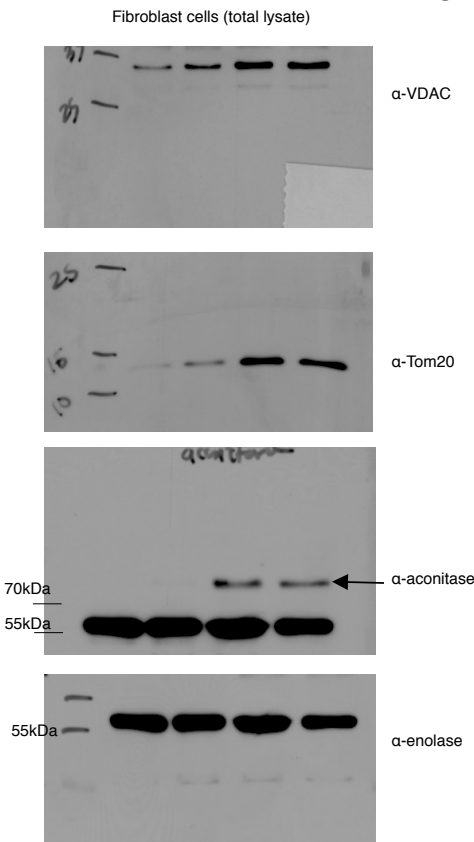

G

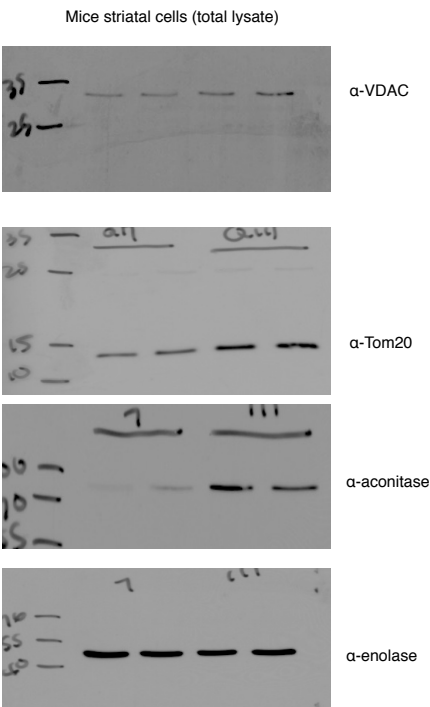

H

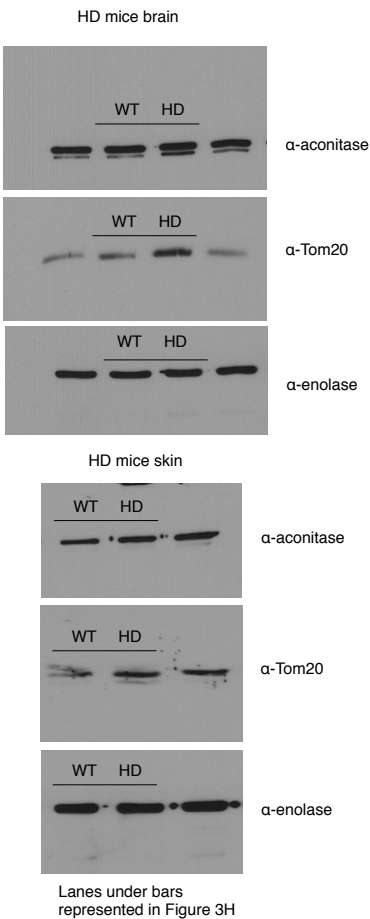

Supplement: Supplementary file 5 [file emmm0007-1307-sd5.pdf]

Figure 4

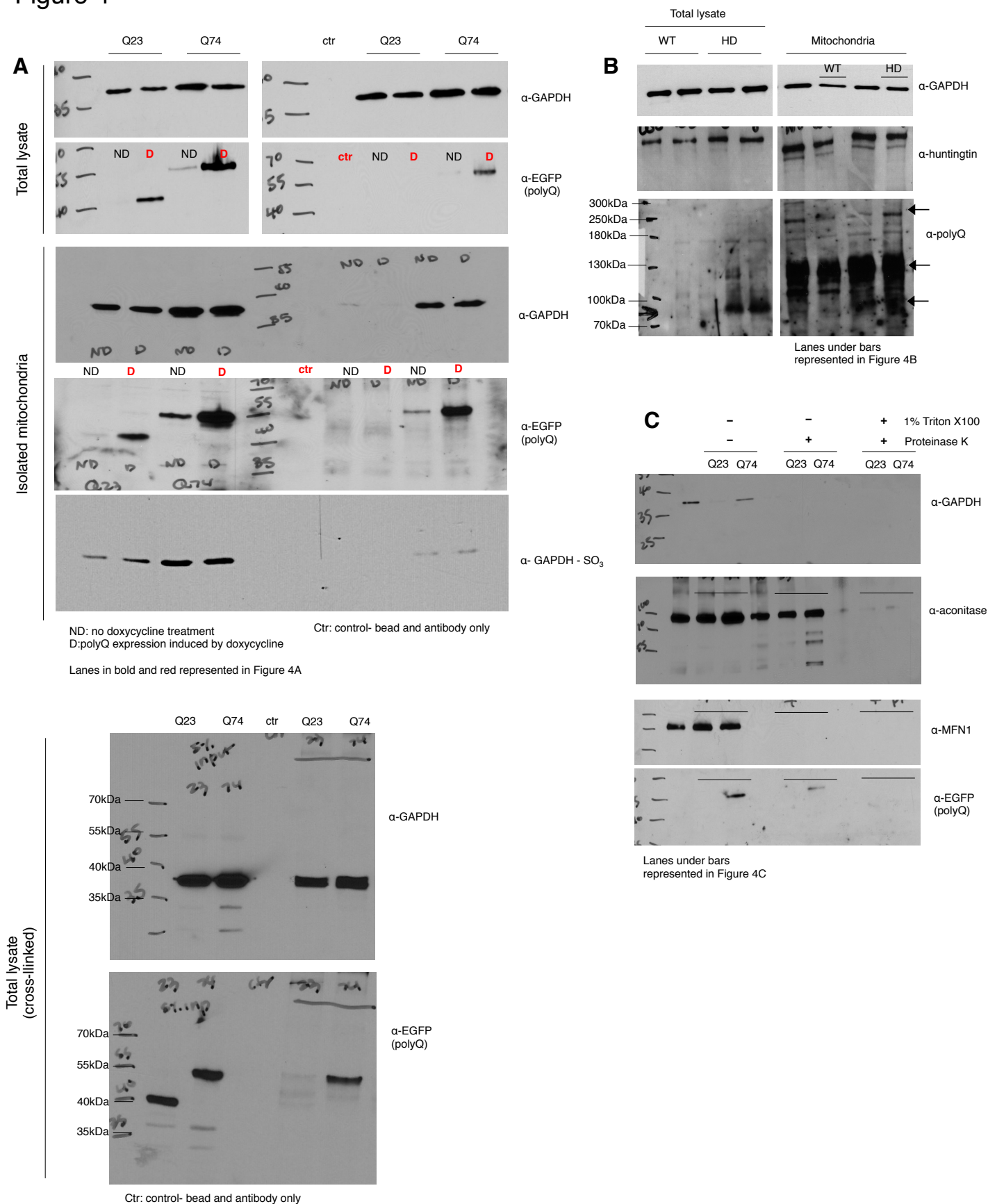

Supplement: Supplementary file 6 [file emmm0007-1307-sd6.pdf]

Figure 5

A

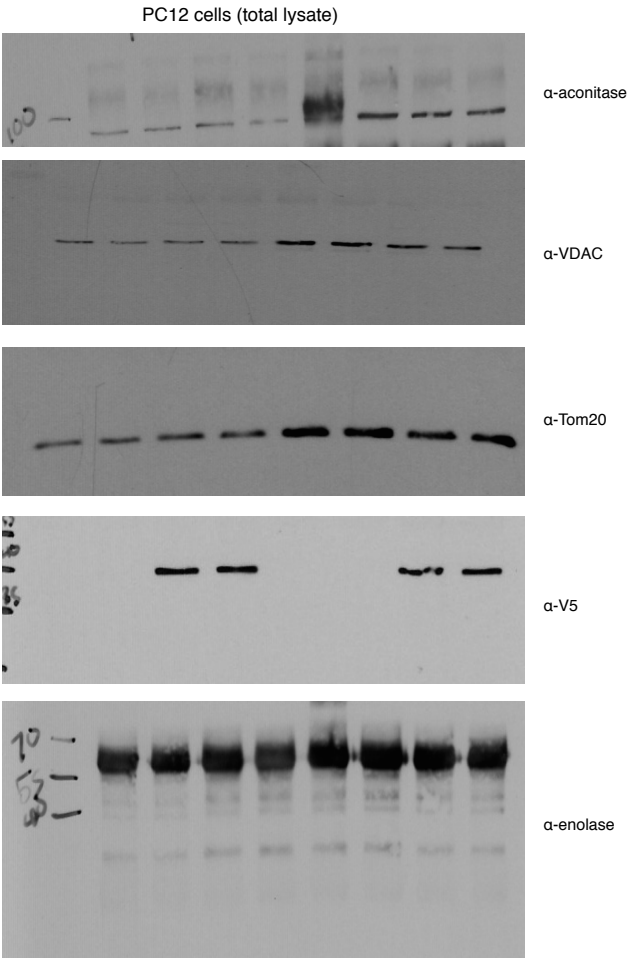

B

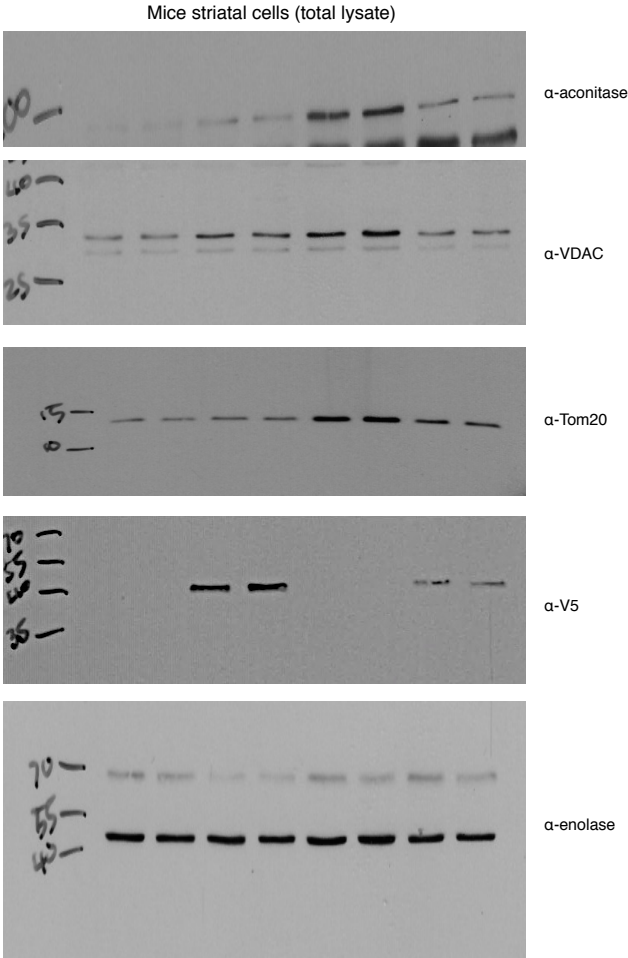

Figure 5

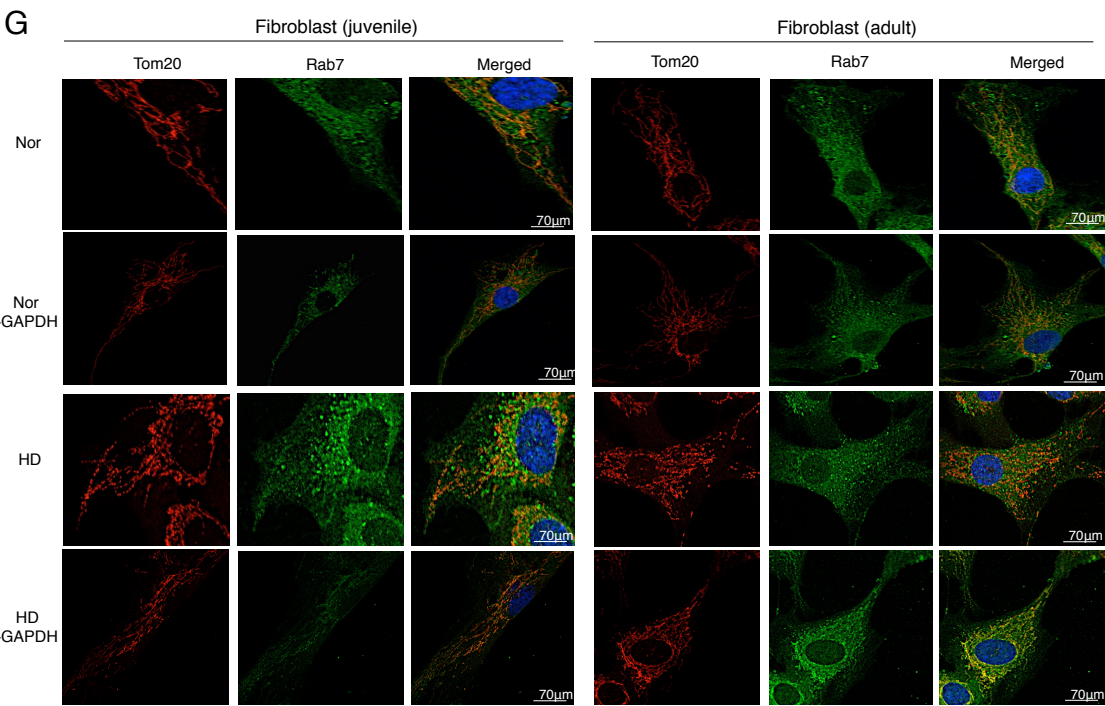

Supplement: Supplementary file 7 [file emmm0007-1307-sd7.pdf]

Figure 6

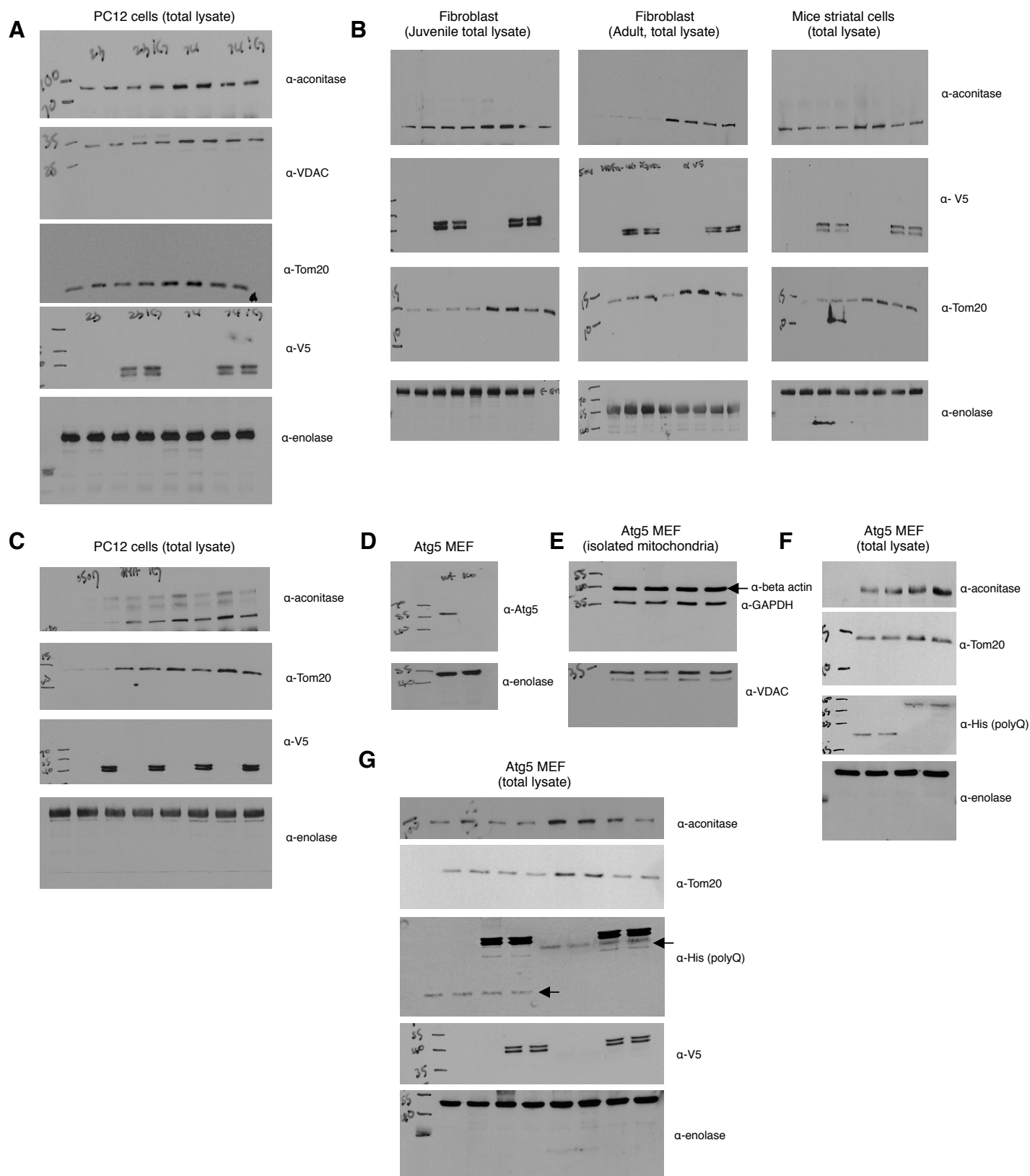

Supplement: Supplementary file 8 [file emmm0007-1307-sd8.pdf]
